# *N* ^*6*^-Methyladenosine-Related Long Non-Coding RNAs Are Identified as a Potential Prognostic Biomarker for Lung Squamous Cell Carcinoma and Validated by Real-Time PCR

**DOI:** 10.3389/fgene.2022.839957

**Published:** 2022-06-03

**Authors:** Wei Zhang, Qian Zhang, Zhefan Xie, Li Che, Tingting Xia, Xingdong Cai, Shengming Liu

**Affiliations:** ^1^ Department of Pulmonary and Critical Care Medicine, The First Affiliated Hospital of Jinan University, Guangzhou, China; ^2^ Department of Pulmonary and Critical Care Medicine, The Third Affiliated Hospital of Zunyi Medical University (The First People’s Hospital of Zunyi), Zunyi, China; ^3^ Department of Renal Medicine, The First Affiliated Hospital of Jinan University, Guangzhou, China

**Keywords:** lung squamous cell carcinoma, m^6^A, long non-coding RNA, prognostic biomarker, PD-L1, tumor microenvironment

## Abstract

Currently, the precise mechanism by which *N* ^*6*^-methyladenosine (m^6^A) modification of long non-coding RNAs (lncRNAs) promotes the occurrence and development of lung squamous cell carcinoma (LUSC) and influences tumor microenvironment (TME) remains unclear. Therefore, we studied the prognostic value of m^6^A-related lncRNAs and their relationship with TME in **495** LUSC samples from The Cancer Genome Atlas (TCGA) database. Pearson’s correlation and univariate Cox regression analysis identified 6 m^6^A-related lncRNAs with prognostic values for LUSC patients. LUSC patients were divided into two subgroups (clusters 1 and 2) using principal component analysis. The expression of PD-L1 was lower in tumor tissues and cluster 2 of LUSC patients. Cluster 2 of LUSC patients had a high immune score, stromal score, and unique immune cell infiltration. The focal adhesion kinase (FAK) pathway and cytokine receptor pathways are enriched in cluster 1. The m^6^A-related lncRNA prognostic markers (m^6^A-LPMs) were established using the least absolute shrinkage and selection operator (LASSO) Cox regression analysis. The risk score was calculated by 4 m^6^A-LPMs and associated with OS, TME, clinicopathological characteristics of LUSC patients. After adjusting for age, gender, and stage, the risk score was also an independent prognostic factor for LUSC patients. Real-time PCR results showed that the expression of 4 m^6^A-LPMs was consistent with our prediction results. Our study found that 4 m^6^A-LPMs (AC138035.1, AC243919.2, HORMAD2-AS1, and AL122125.1) are closely associated with LUSC prognosis, in future, they may as novel diagnostic biomarkers for LUSC and provide new immunotherapy targets for LUSC patients.

## 1 Introduction

Lung cancer is currently the second leading cause of global cancer incidence and the primary cause of cancer death in the worldwide. In 2020, about 2.2 million new lung cancer cases were diagnosed, and another 1.8 million died from lung cancer worldwide (Sung et al., 2021). Non-small cell lung cancer (NSCLC) is the most common type of lung cancer, accounting for about 85%, which mainly includes lung adenocarcinoma (LUAD) and lung squamous cell carcinoma (LUSC) (Woodman et al., 2021). Although significant progress has been made in lung cancer treatment modalities, including surgery, radiotherapy, chemotherapy, and targeted therapy, the 5-years survival rate of NSCLC patients is still low, at about 16.6% (Torre et al., 2016; Liu et al., 2020). Various factors determine the development and progression of NSCLC. The genetic makeup of tumor cells and the tumor microenvironment (TME) have critical regulatory roles (Forde et al., 2014). The immune system, in particular, plays a dual role in the evolution of tumors. It can identify and control new tumor cells through immune surveillance and promote tumor progression through various mechanisms of immunosuppression (Kunimasa and Goto, 2020). As a result, immunotherapy has gradually shifted from second-line therapy to first-line therapy since 2013, and patients without targeted oncogene mutations have benefited from immunotherapy (Zhang et al., 2019). Paclitaxel and carboplatin combined with immune checkpoint inhibitors such as pembrolizumab is the first-line treatment for LUSC at present (Fan et al., 2019; Lin et al., 2020). This method can significantly prolong the overall survival and progression-free survival of LUSC patients (Paz-Ares et al., 2018). However, the long-term follow-up shows that patients treated with immunotherapy may eventually develop resistance to immunotherapy, and in some patients tumor progression may even be accelerated after immunotherapy (Huang et al., 2015; Ribas, 2015), which may be related to the imbalance of the immune system in LUSC patients. Therefore, it is necessary to investigate the regulatory network of the immune TME to identify new biomarkers and targets for the prognosis and immunotherapy of patients with LUSC.

N^6^-methyladenosine (m^6^A) methylation is one of the most common post-transcriptional modifications. It is reported to play an essential role in normal physiological processes and pathological processes leading to various diseases (Ma et al., 2019). M^6^A methylation modification is the methylation of the sixth N atom of adenine (A) in RNA, referred to as m^6^A, which mainly occurs in mRNA and accounts for about 80% of all RNA methylation (Ping et al., 2014). Recent studies have found that m^6^A modifications can also regulate the generation and function of non-coding RNA, such as micro-RNAs (miRNAs), long non-coding RNAs (lncRNAs), and circular RNAs (circRNAs) (Yang et al., 2017; Wu et al., 2018; Yang et al., 2018). The m^6^A modification of RNA is a dynamic and reversible mode of regulation. It is co-regulated by m^6^A methyltransferase (writers) and demethylase (erasers), and can be selectively recognized and bound by m^6^A recognition proteins (readers) to regulate gene expression post-transcription (Zaccara et al., 2019). Some studies have found that m^6^A promotes tumor progression by upregulating oncogenes in various tumors (He et al., 2019). Liu et al. (2020) performed a reliable cluster analysis on the gene expression of 13 m^6^A-related proteins and divided LUAD patients into two groups with significant differences in age, race, tumor size, stage, and distant metastasis, and their expression profile analysis showed that the expression of m^6^A-related genes could be used as the prognostic criterion for patients with advanced LUAD. Some studies have also found that the m^6^A demethylase FTO promotes the tumor progression of LUSC by regulating the expression of MZF1 and provides a potential target for LUSC treatment (Liu et al., 2018). These findings indicate that m^6^A methylation plays a vital role in the progression and prognosis of LUSC.

LncRNAs are a class of non-coding RNAs with a length of more than 200 nucleotides, which do not encode proteins but have some characteristics of mRNA (Cabili et al., 2011). Their abnormal expression in lung cancer can promote tumor progression. For example, MALAT1, which has carcinogenic effects, is the commonest lncRNA in lung cancer, which can promote NSCLC tumor growth and metastasis by regulating the miR124/STAT3 and miR204/SLUG axis (Li et al., 2016; Li et al., 2018). The interaction between m^6^A and lncRNAs has two modes: lncRNAs regulating m^6^A-modified RNAs and m^6^A-modified lncRNAs (Dai et al., 2020). M^6^A has extensive modifications to lncRNAs that functionally regulate the eukaryotic transcriptome, affecting RNA splicing, export, localization, translation, and stability (Linder et al., 2015). Some studies have found that m^6^A modification can regulate the stability or expression of lncRNAs, which regulate tumor progression through the lncRNA-mediated ceRNA network (Fazi and Fatica, 2019; Chen J. et al., 2020; Huang et al., 2020). For instance, there is a triple helix structure at the 3 ‘terminal of lncRNA MALAT-1, and the deletion or mutation of this structure affects the stability and expression of MALAT1 (Brown et al., 2014). Methyltransferase METTL16 can bind to the triple helix structure at the 3 ‘terminal of lncRNA MALAT1 and influence its structural stability and functional expression, thus participating in the occurrence and development of tumor (Brown et al., 2016). Studies have reported that METTL3 can improve the methylation level of m^6^A and enhance the transcriptional stability of lncRNA ABHD11-AS1 to increase its expression. At the same time, the overexpression of METTL3 is closely related to the poor prognosis of patients with NSCLC (Xue et al., 2021). In recent years, some studies have also found that m^6^A-related lncRNAs are potential biomarkers for predicting the prognosis and immune response in LUAD patients (Xu et al., 2021; Zheng et al., 2021). However, the mechanism how the lncRNAs modificated by m^6^A promote the occurrence and development of LUSC remains unclear. Therefore, to understand the regulating role of m^6^A modificated lncRNAs in the progression of LUSC may help us to get new biomarkers and valuable therapeutic targets.

**FIGURE 1:**
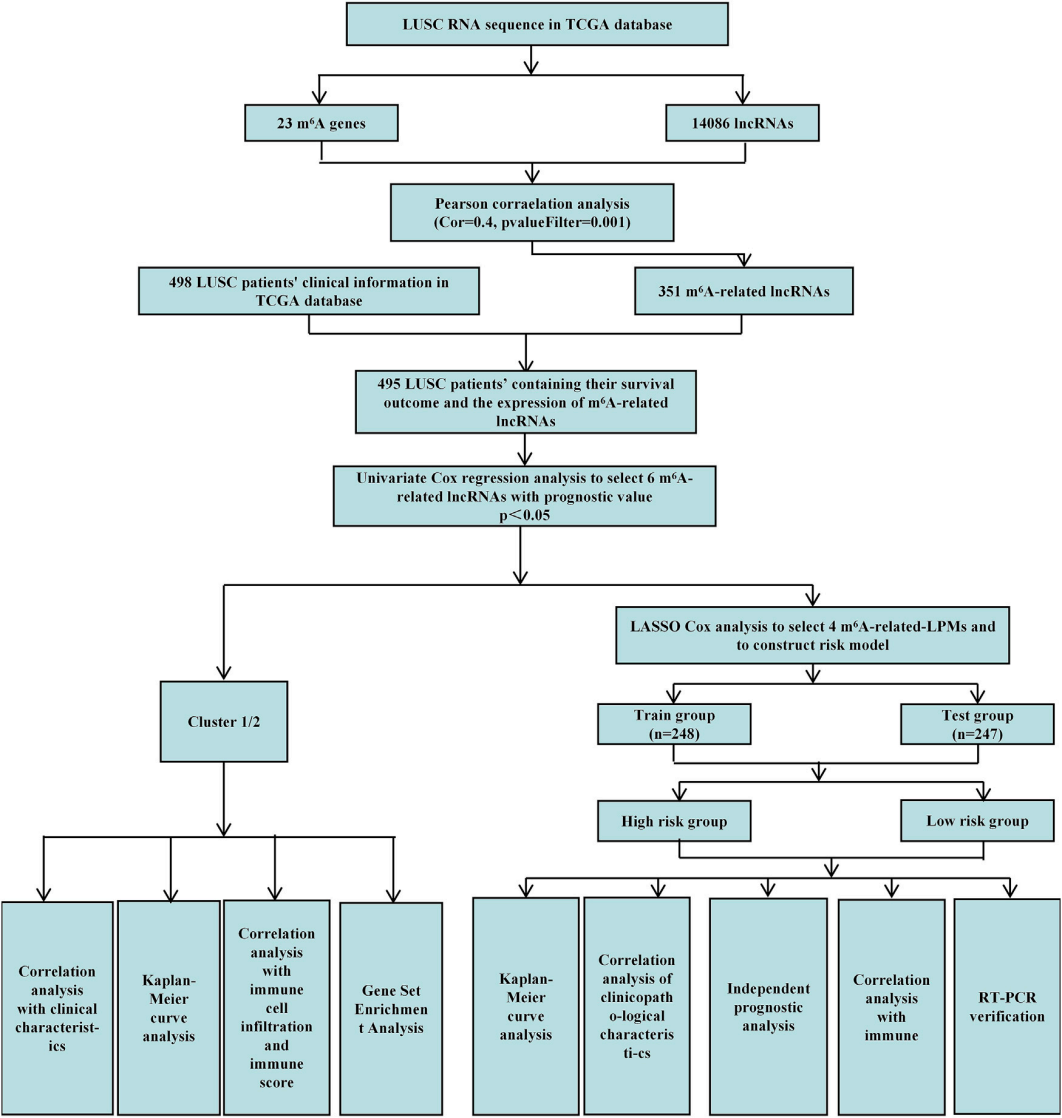
Study flowchart of this research.

This study uses Pearson correlation analysis to identify potential m^6^A-related lncRNAs. Then, univariate Cox regression analysis identified m^6^A-related lncRNAs with prognostic values. We analyzed their expression in lung tumor tissues and normal tissues datasets through bioinformatics and statistical methods. We also established cluster subtypes of m^6^A-related lncRNAs with prognostic values and systematically assessed the correlation between these lncRNAs in different clusters with clinicopathological characteristics, prognosis, PD-L1, and TME. Gene enrichment analysis was performed to evaluate the possible reasons for the differences in TME of the different clusters. Additionally, we identified 4 m^6^A-related lncRNA prognostic markers (m^6^A-LPMs) based on the prognostic value of m^6^A-related lncRNAs using the LASSO Cox analysis. We randomly divided 495 LUSC patients into the training and test groups. In addition, we constructed a risk model and calculated the risk score of each patient based on the expression and regression coefficients of the four m^6^A-LPMs in the different groups. According to the median risk score, all patients were divided into high-risk and low-risk groups. We evaluate the relationship between the clinicopathological characteristics, prognosis, immune score, and immune cell infiltration in the high- and low-risk groups. Real-time PCR was used to further verify the expression of m^6^A-LPMs in LUSC cell line HCC1588 and human bronchial epithelial cell line 16HBE. The Study flowchart of this research is shown in Figure 1.

## 2 Materials and Methods

### 2.1 Datasets and m^6^A-Related Genes

The RNA-seq transcriptome data and related clinical data of LUSC patients were downloaded from the TCGA data portal (https://portal.gdc.cancer.gov/). Data from 504 LUSC samples and 49 adjacent normal tissues were downloaded from the TCGA database. After excluding samples with unknown survival time and survival status from clinical features, we ultimately obtained 498 LUSC patient samples and their corresponding clinicopathological information, including survival time, survival status, age, sex, and TNM staging for further analysis. We extracted 23 m^6^A-related gene expression matrices from the TCGA dataset based on the data in previous publications, including the expression data of writers (METTL3, METTL14, METTL16, WTAP, VIRMA, ZC3H13, RBM15, and RBM15B), readers (YTHDC1, YTHDC2, YTHDF1, YTHDF2, YTHDF3, HNRNPC, FMR1, LRPPRC, HNRNPA2B1, IGFBP1, IGFBP2, IGFBP3, RBMX) and erasers (FTO and ALKBH5) (Wojtas et al., 2017; Tang et al., 2018; Yu et al., 2021; Wang et al., 2021; and Xu et al., 2021.).

### 2.2 Annotation of lncRNAs

Obtain The annotation file of the lncRNAs of the Genome Reference Consortium Human Build 38 (GRCh38) from the GENCODE website (https://www.gencodegenes.org/human) to annotate the lncRNAs in the TCGA database. 14,086 lncRNAs were identified in the LUSC datasets from the TCGA database by identifying the Ensemble IDs of the gene.

### 2.3 Identification of m^6^A-Related lncRNAs and Construction of a Co-expression Network

We acquired detailed clinical information for **498** LUSC patients from the extracted clinical data in the TCGA database. We assessed survival outcomes of the LUSC patients based on the overall survival (OS). Through the “limma” package (version 3.42.2) of the R language, we use Pearson correlation analysis to identify potential m^6^A-related lncRNAs based on a correlation coefficient |R| = 0.4 (*p* = 0.001). Then, the “igraph” R package was used to draw a co-expression network of the m^6^A-related lncRNAs and m^6^A-related genes (Figure 1).

### 2.4 Screening of Prognostic-Related lncRNAs and Their Expression Heatmap

First, the “limma” R package (version 3.42.2) merged the expression of m^6^A-related lncRNAs with the survival time and status of 498 LUSC patients. We obtained the survival outcomes and the expression of m^6^A-related lncRNAs of 495 LUSC patients. Then, univariate Cox regression analysis identified m^6^A-related lncRNAs with prognostic values (*p* < 0.05) (Figure 1). Finally, the “pheatmap,” “reshape2,” and “ggpubr” R packages (versions 1.0.12, 1.4.4, and 0.4.0) were used to draw the expression heatmap of prognostic-related lncRNAs in lung cancer tumor tissues and normal tissues. We also classified the LUSC patients into different groups using the “limma” and the “ConsensusClusterPlus” R packages (versions 3.42.2 and 1.58.0) based on the expression levels of m^6^A-related lncRNAs with prognostic values.

### 2.5 Survival Analysis and Clinical Correlation Analysis Between Clusters 1 and 2

We used the “survival” and “survminer” R packages (versions 3.2–11 and 0.4.9) to compare survival outcomes between the two clusters of LUSC patients. Then, the “pheatmap” R package (version 1.0.12) drew expression heatmaps of different LUSC clusters and clinical features.

### 2.6 Comparison of Immune Score, Stromal Score, and TME Between the Two Clusters

The immune score of each patient with LUSC was calculated using the estimation algorithm in the “estimate” R package (version 1.0.13). Then, we estimated the cell type identification of the relative subset of RNA transcripts and obtained the ratio of 22 immune cell types for each sample using the “e1071”, “preprocessCore” packages (versions 1.7-6 and 1.48.0), and " CIBERSORT” R script (version 1.03). In this process, we used 1,000 sorting algorithms, and only performed follow-up analysis on samples with a CIBERSORT value < 0.05, and compared the differential immune penetration levels between two clusters.

### 2.7 Gene Set Enrichment Analysis

The differences in TME between two clusters of LUSC were analyzed using the gene set enrichment analysis (GSEA). *p* < 0.05 and a false discovery rate of <0.05 were considered statistically significant.

### 2.8 Construction of m^6^A-Related lncRNA Prognostic Markers

The 495 LUSC patients were randomly divided into the training group (248 patients) and the test group (247 patients) with a 1:1 ratio by using the “caret” R package (version 6.0–86). We established 4 m^6^A-related lncRNA prognostic markers (m^6^A-LPMs) by Least Absolute Shrinkage and Selection Operator (LASSO) Cox regression analysis (using 10-fold cross-validation to estimate the penalty parameters) using the “glmnet” R package (version 4.1–1). Using the coefficients generated by the LASSO regression algorithm, we obtained the risk score equation: risk score = sum of coefficients×m^6^A-related-LPMs expression level, which was used to calculate the risk score of each patient in the training and test group. Then, the median of the risk score was set as the standard, and the patients were divided into high-risk and low-risk groups (Figure 1).

### 2.9 Survival Analysis Between High-Risk and Low-Risk Groups

According to the risk score, using the “survival,” “survminer,” “timeROC,” and “pheatmap” R packages (versions 3.2–11, 0.4.9, 0.4 and 1.0.12) to compare survival outcomes between high-risk and low-risk groups.

### 2.10 Correlation Analysis of the Risk Score and Clinicopathological Characteristics

The risk scores and clinicopathological characteristics of all patients were integrated using the “survival,” “survminer,” and “pheatmap” R packages (versions 3.2–11, 0.4.9, and 1.0.12) to draw heatmaps and survival curves of the correlation between different risk scores and clinicopathological characteristics.

### 2.11 Independent Prognostic Analysis

After integrating the risk scores of the training group and the test group with clinical data, we used univariate and multivariate Cox regression analysis to assess whether the risk score can be an independent prognostic factor for LUSA patients. The “survival” R package (version 3.2–11) was used to draw a forest map of Cox regression analysis.

### 2.12 Correlation Analysis Between Risk Score and Immunity

We used the “limma,” “ggplot2”, “ggpubr,” and “ggExtra” R packages (versions 3.42.2, 3.3.5, 0.4.0, and 0.9) to integrate the risk score and the content of immune cells in order to analyze the correlation between the risk score and the expression level of immune cells.

### 2.13 Cell Culture

The human bronchial epithelial cell line 16HBE was cultured in Dulbecco’s modified Eagle medium (DMEM, Gibco), and LUSC cell line HCC1588 was cultured in RPMI1-1,640 (Solarbio), which contained 10% heat-inactivated fetal bovine serum (FBS) and 1% penicillin/streptomycin. All cells are incubated in an incubator containing 5% CO_2_ at 37°C.

### 2.14 16HBE Cells and HCC1588 Cells RNA Extraction and Real-Time PCR

The expression of lncRNAs was detected by real-time PCR in different cell lines. Using Trizol method to extract total RNA from different cells. Primers for AC138035.1 (forward, 5′-TCA​GAT​GAG​CAG​CAG​CGT​TAG​ATT​C-3′, reverse, 5′- GAG​CGT​GAT​GGG​AGA​AAG​TGA​CAG-3′), Primers for HORMAD2-AS1 (forward, 5′-GCA​GGA​GAA​CCA​CAG​TGA​CAA​CC-3′, reverse, 5′- TGC​TGA​CAG​TAG​TGC​TTG​CCT​ATT-3′), Primers for AC243919.2 (forward, 5′-CTC​GCC​AAC​TGG​TCC​TTC​ATC​TTC-3′, reverse, 5′- CTC​CTT​CAT​GCT​AAG​CCT​CCT​CTT​G-3′), Primers for AL122125.1 (forward, 5′-CAA​GCA​TGT​GGC​ACT​AGA​GGA​GAC-3′, reverse, 5′- CAG​AGA​TGG​AGG​CAG​AGG​TTG​AAT​G-3′) and Primers for GAPDH (forward, 5′-TGA​CTT​CAA​CAG​CGA​CAC​CCA-3′, reverse, 5′- CAC​CCT​GTT​GCT​GTA​GCC​AAA-3′) by real-time PCR and it was performed with three replicates of samples from three independent experiments.

### 2.15 Statistical Analysis

In this study, the R programming language (version 3.6.1, http://www.R-project.org) was used for analysis. The Kaplan-Meier method was used to generate the survival curves, and the log-rank test compared the differences between groups. The receiver operating characteristic (ROC) curves were used to estimate the predictive efficiency of m^6^A-LPMs for the 1-year overall survival (OS). Real-time PCR experimental data are expressed as the mean ± standard deviation (SD) of at least three independent experiments. All statistical analysis was performed using GraphPad Prism 8 (GraphPad Software, La Jolla, CA, USA). The student’s t-test (two-tailed) compares the two groups in real-time PCR. A *p* < 0.05 indicates statistical significance.

## 3 Results

### 3.1 Clinical Characteristics of LUSC Patients

We obtained prognostic information and clinicopathological features in a total of 498 LUSC patients after excluding patients with unknown survival outcomes. The clinicopathological characteristics of the patients are shown in Table 1.

**TABLE 1:** Clinicopathological characteristics of LUSC patients in the TCGA cohort.

		LUSC	
		Number	Percentage
Total		498	100
Age	33–90		
	≤65	189	37.95%
	>65	303	60.84%
	unknown	6	1.21%
Gender			
	Female	130	26.10%
	Male	368	73.90%
Stage			
	Stage I	243	48.80%
	Stage II	160	32.13%
	Stage III	84	16.87%
	Stage IV	7	1.40%
	unknown	4	0.80%
Tumor (T)			
	T1	114	22.89%
	T2	290	58.23%
	T3	70	14.06%
	T4	24	4.82%
Lymph Node (N)			
	N0	317	63.65%
	N1	130	26.10%
	N2	40	8.03%
	N3	5	1.01%
	NX	6	1.21%
Metastasis (M)			
	M0	410	82.33%
	M1	7	1.41%
	MX	77	15.46%
	unknown	4	0.80%

LUSC, lung squamous cell carcinoma.

### 3.2 Prognosis of m^6^A-Related lncRNAs in LUSC Patients

**FIGURE 2:**
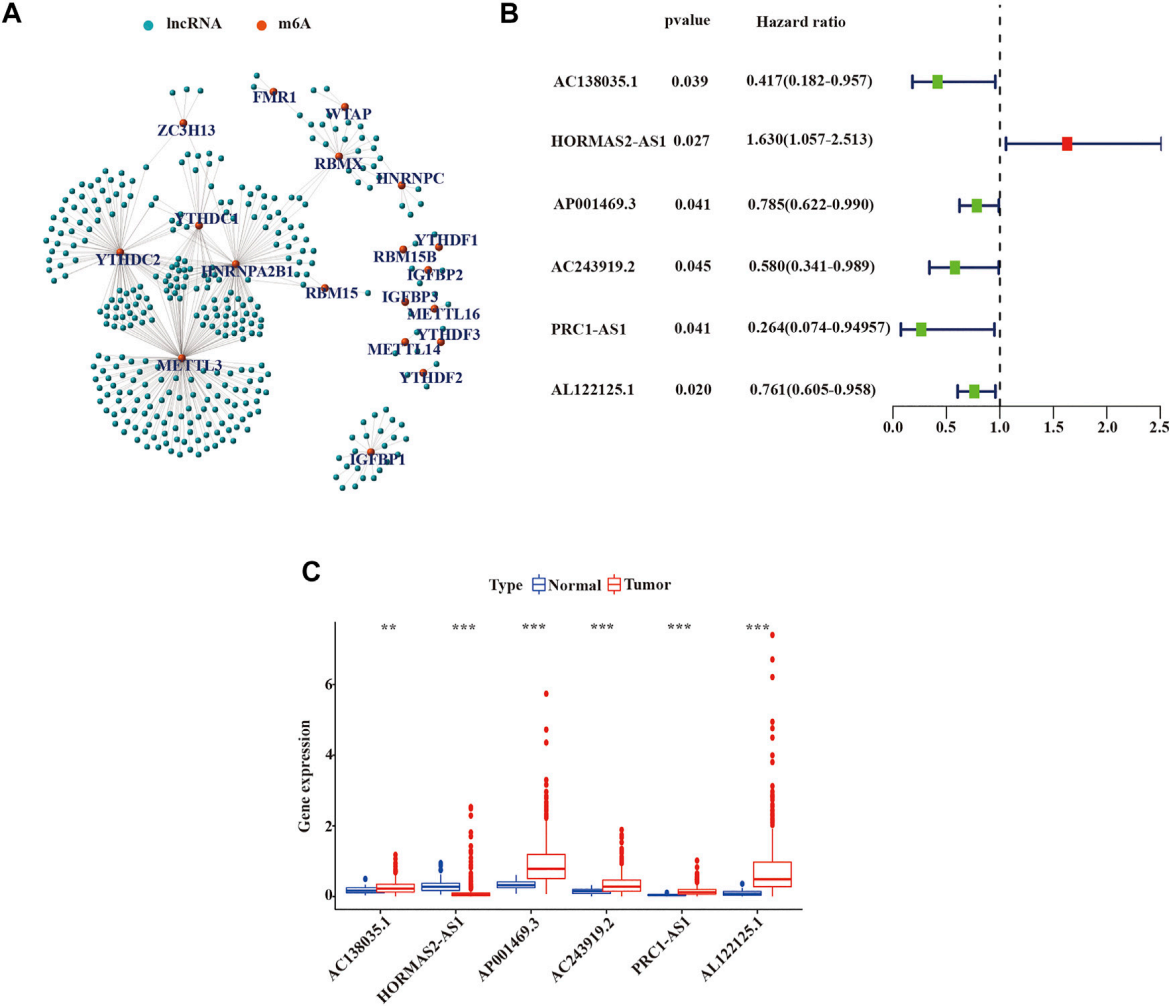
Identification of m^6^A-related lncRNAs with prognostic value in LUSC patients. **(A)** Co-expression network of m^6^A-related genes and m^6^A-related lncRNAs. **(B)** m^6^A-associated lncRNAs with prognostic values were validated by univariate Cox analysis. **(C)** Expression of 6 prognostic m6A-related lncRNAs in tumor and normal tissues. LUSC, lung squamous cell carcinoma; m^6^A, *N* ^*6*^-methyladenosine; lncRNAs, long non-coding RNAs; Statistical methods: Wilcoxon test **p < 0.01 and ***p < 0.001.

We defined lncRNAs with expression levels that correlated with one or more of the 23 m^6^A-related genes as m^6^A-related lncRNAs by Pearson’s correlation analysis, according to the selection criteria R > |0.4|, *p* = 0.001. We identified that the expression of 351 lncRNAs was significantly correlated with m^6^A-related genes and constructed the co-expression network of m^6^A regulatory genes and m^6^A-related lncRNA (Figure 2A and Supplementary Tables S1, S2). Subsequently, we combined the expression levels of **351** m^6^A-related lncRNAs with the survival period and status of **498** LUSC patients to identify lncRNAs with prognostic value. Univariate Cox regression analysis was used to analyze prognostic information and expression of m^6^A-related lncRNAs in the TCGA database (*p* < 0.05). We finally identified the expression of 6 m^6^A-related lncRNAs: AC138035.1, AP001469.3, AC243919.2, PRC1−AS1, AL122125.1, and HORMAD2−AS1 was significantly impact on the OS of LUSC patients. (Figure 2B and Supplementary Table S3). Meanwhile, we analyzed the expression of prognostic m^6^A-related lncRNAs in LUSC tissues and normal tissues. As shown in Figure 2C, an increased expression of AC138035.1, AP001469.3, AC243919.2, PRC1−AS1, and AL122125.15 were observed in LUSC tissues compared to normal tissues.

### 3.3 Consensus Clustering of m^6^A-Related lncRNAs in LUSC Patients

**FIGURE 3:**
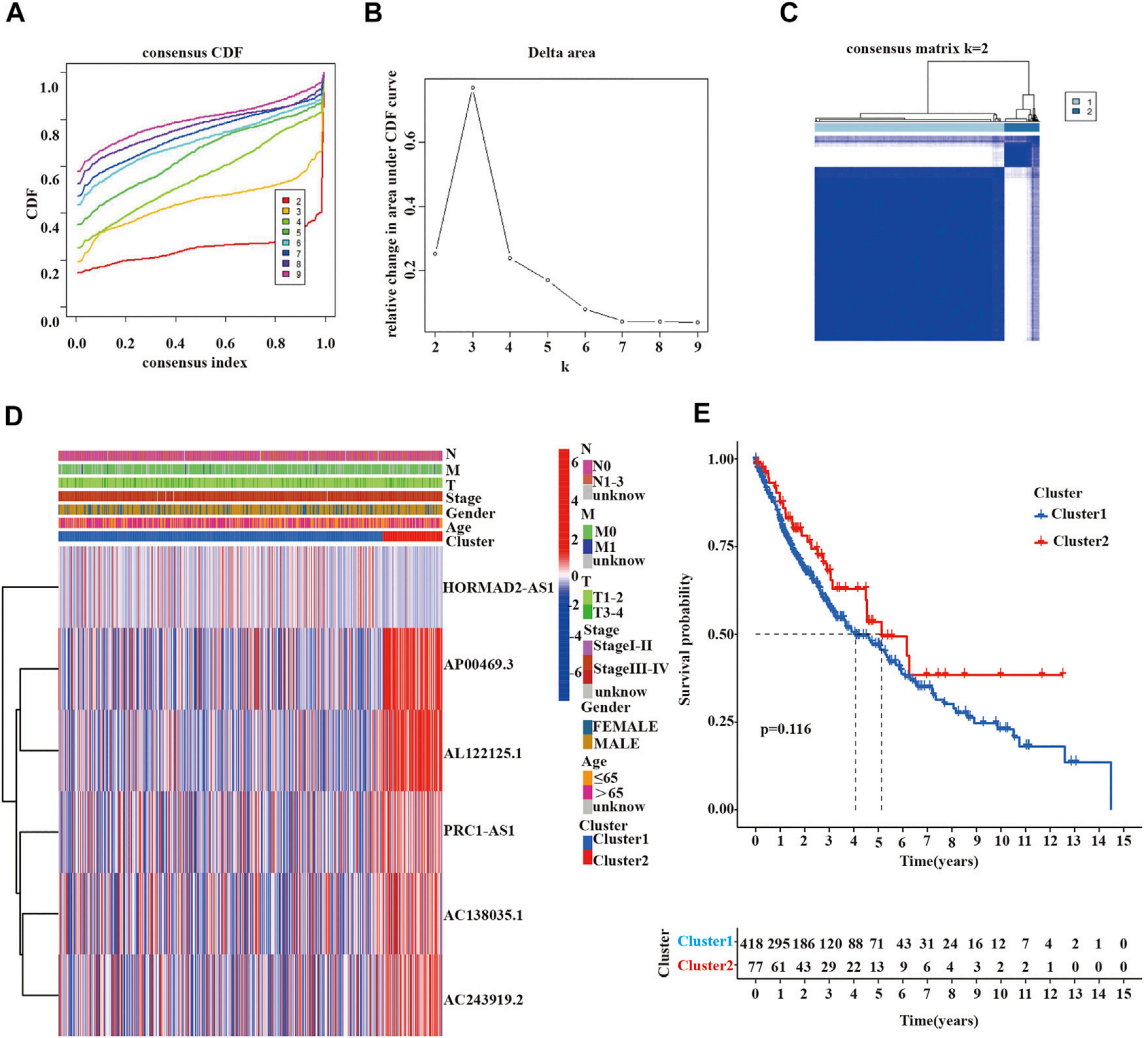
Clinicopathological characteristics and survival analysis of cluster 1/2 subgroups of LUSC. **(A,B)** Consensus clustering cumulative distribution function (CDF) for k = 2 to 9. **(C)** Consensus clustering matrix for k = 2. **(D)** Heatmap of the correlation between the expression levels of 6 m^6^A-related lncRNAs with prognostic value and clinicopathological features in the TCGA-LUSC dataset (red indicates high expression, blue indicates low expression). **(E)** Kaplan-Meier curves of overall survival for two clusters in LUSC. LUSC, lung squamous cell carcinoma; m^6^A, *N* ^*6*^-methyladenosine; lncRNAs, long non-coding RNAs; TCGA, the cancer genome atlas; Statistical methods: Chi-square test, log-rank test.

To further estimate the clinical relevance of m^6^A-related lncRNAs with prognostic value in LUSC patients, we used the “ConsensusClusterPlus” R package to divide LUSC tissue samples based on the expression of prognostic lncRNAs into two subgroups. In consensus clustering analysis, a k represented the cluster count, and k = 2-9 in the cumulative distribution function (CDF) (Figure 3A). The stability of consensus clustering analysis results should meet three conditions: the curve area does not increase significantly under the CDF; there was a close correlation among different clusters; the number of samples in a particular cluster cannot be too small. Figure 3B showed that the CDF curves of the consensus matrix did not significantly increase when k = 2. In addition, **we** selected k = 2 as the consensus matrix k value to divide the LUSC cohorts into two independent subgroups, cluster 1 (n = 418) and cluster 2 (n = 77); there was a significant correlation within the same cluster and a very low correlation between different clusters (Figure 3C). Heat maps showed the expression of 6 prognostic m^6^A-related lncRNAs in different clusters and their relationship with clinicopathological features (Figure 3D). We found no statistical significance among the two clusters in OS of LUSC patients (Figure 3E).

**FIGURE 4:**
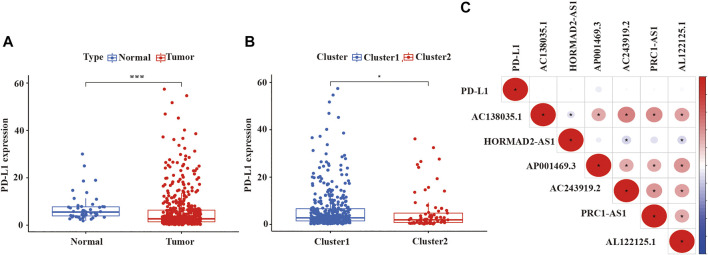
The expression level of PDL-1 in LUSC and the relationship between PD-L1 with prognostic-related lncRNAs. **(A)** The expression of PDL-1 in LUSC tissue samples. **(B)** The expression of PDL-1 in two clusters. **(C)** The relationship between PDL-1 and prognostic-related lncRNAs. LUSC, lung squamous cell carcinoma; lncRNAs, long non-coding RNAs; PD-L1, programmed cell death 1 ligand 1. Statistical methods: Wilcoxon test. *p < 0.05, ***p < 0.001.

Because previous studies revealed that multiple lncRNAs indirectly regulated PD-L1 expression to impact the survival of cancer patients, immune checkpoint inhibitors (ICIs) targeting programmed death-1/programmed death ligand 1 (PD-1/PD-L1) had been integrated into standard-of-care regimens for patients with advanced LUSC (Tian P. et al., 2021; Tian Y. et al., 2021; Mu et al., 2021; and; Yuan et al., 2021). Some studies also reported that the effect of first-line treatment with the PD-1 inhibitor pembrolizumab was superior to platinum-doublet chemotherapy in patients with non-small-cell lung cancer and high PD-L1 expression (Aguilar et al., 2019). Therefore, we wanted to estimated the expression of PD-L1 among different clusters and which cluster would more benefit from pembrolizumab, a PD-1 inhibitor. As shown in Figure 4, we detected that the expression of PD-L1 in LUSC tissue samples was lower than normal tissue samples (*p* < 0.001, Figure 4A), as well as the expression of PD-L1 in cluster 2 was also lower than cluster 1 (*p* < 0.05, Figure 4B). However, the expression of PD-L1 was not significantly correlated with the expression of lncRNA in LUSC (Figure 4C).

### 3.4 Immune Score, Stromal Score, and TME in Different Clusters

**FIGURE 5:**
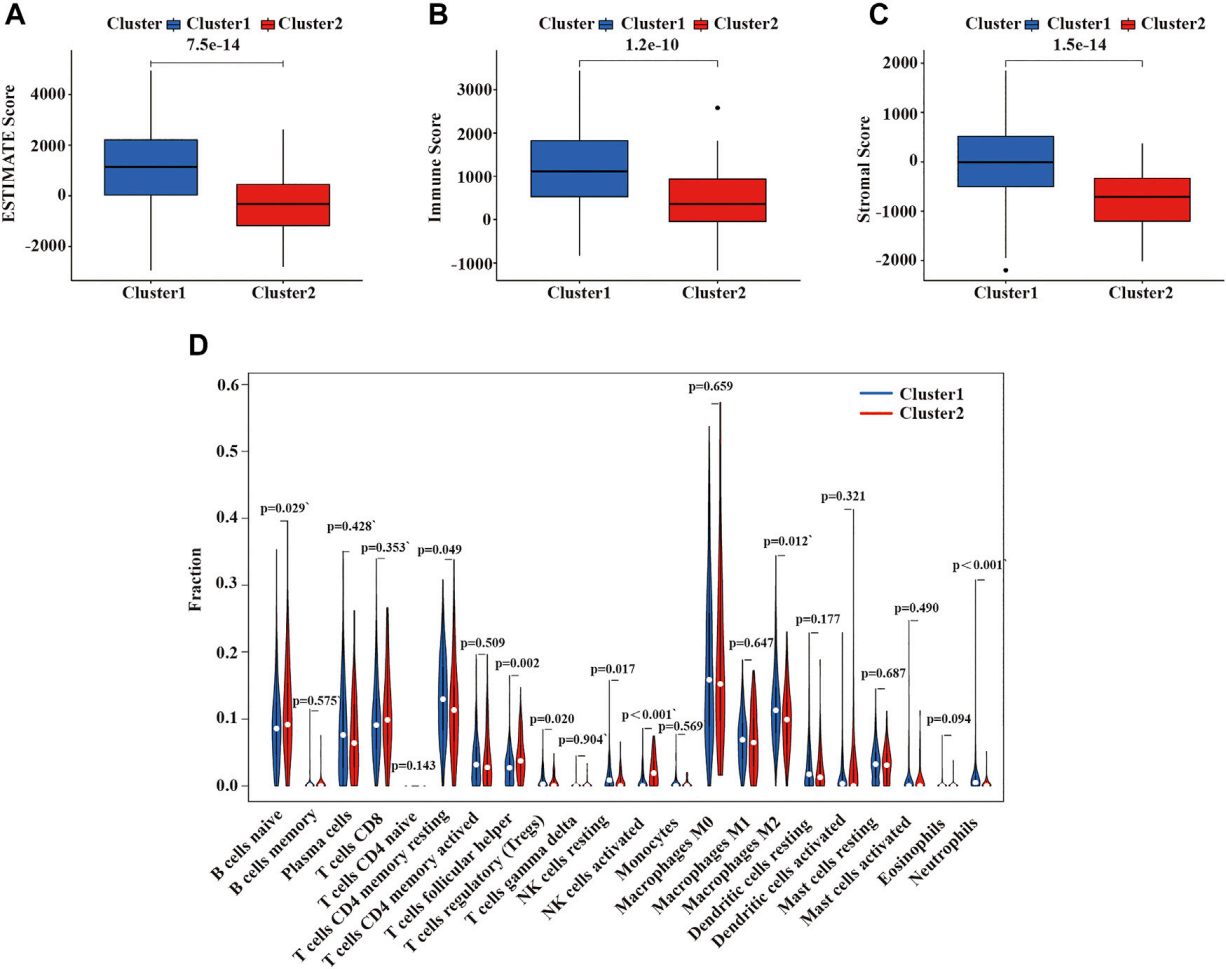
Immune function and immune cell infiltration in the two clusters of LUSC. **(A)** ESTIMATE score in the two clusters. **(B)** Immune score in the two clusters. **(C)** Stroma score in the two clusters. **(D)** The infiltration level of 22 immune cells in the two clusters. LUSC, lung squamous cell carcinoma. Statistical methods: Wilcoxon test.

Malignant solid tumor tissue is composed of tumor-associated epithelial cells, immune cells, stromal cells, vascular cells, and tumor cells (Yoshihara et al., 2013). Stromal cells play an essential role in tumor progression and drug resistance, and infiltrating immune cells can regulate the tumor microenvironment (TME) to influence tumor growth, invasion, and metastasis. (Hanahan and Weinberg, 2011; Mlecnik et al., 2011; Straussman et al., 2012,). The stromal score was planned to capture the presence of stroma in tumor tissues, and the immune score was designed to represent the infiltration of immune cells in tumor tissues. The estimated score was inferred tumor purity (Yoshihara et al., 2013). We use the ESTIMATE algorithm to calculate the immune, stromal, and ESTIMATE scores of each patient with LUSC patients. As is shown in Figures 5A–C, the ESTIMATE score, immune score, and stromal score of cluster 2 were lower than cluster 1. Additionally, we also assessed the expression of 22 immune cells in the two clusters of LUSC using the CIBERSORT algorithm. In cluster 1, the numbers of CD4 memory resting T cells, activated M2 macrophages, neutrophils, regulatory T cells (Tregs), and resting NK cells were higher than cluster 2; however, the numbers of naïve B cells, follicular helper T cells, and activated NK cells were higher in cluster 2 compared with cluster 1 (Figure 5D).

### 3.5 GSEA Analysis Between the Two Clusters of LUSC

**FIGURE 6:**
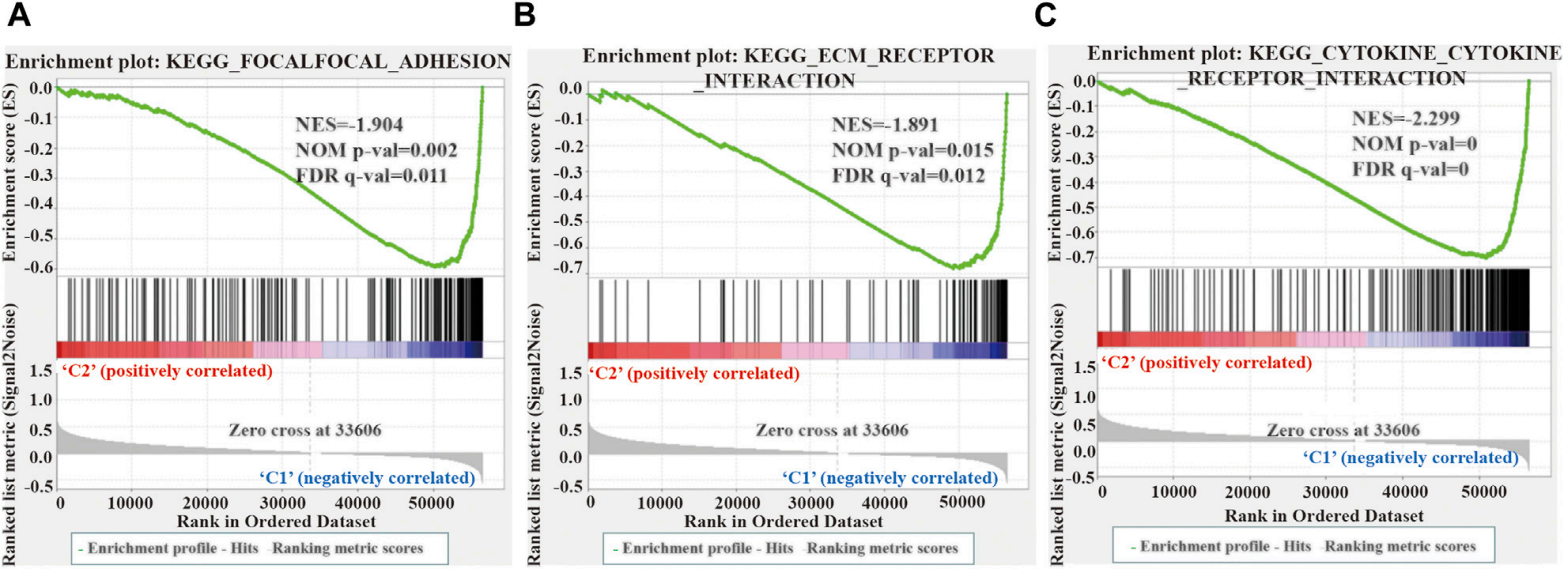
Gene enrichment analysis (GSEA) among different two clusters. **(A–C)** GSEA shows that focal adhesion kinase **(A)**, extracellular matrix receptor interaction **(B)**, and cytokine receptor **(C)** are enriched in cluster 1 of LUSC. LUSC, lung squamous cell carcinoma.

We performed GSEA to elucidate the potential regulatory mechanisms leading to the difference in TME between the two clusters. The enrichment analysis revealed that the malignant tumors correlated pathways, such as focal adhesion kinase (FAK), extracellular matrix (ECM) receptor interaction, and cytokine receptors, were mainly enriched in cluster 1 (Figure 6). Therefore, the activation of the FAK pathway and cytokine receptors may be the main reason for the difference in TME in LUSC cluster 1/2.

### 3.6 Identification and Evaluation Prognosis of 4 m^6^A-LPMs

**FIGURE 7:**
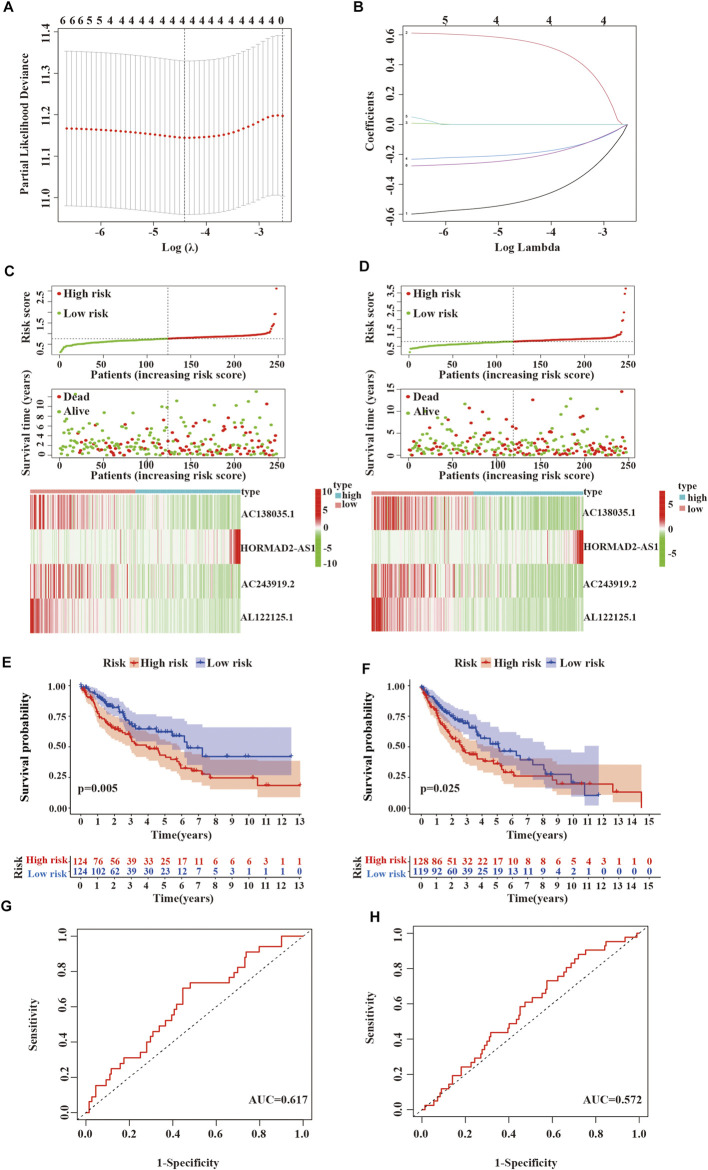
Verifying the risk model. **(A,B)** LASSO Cox regression analysis of 6 m^6^A-related lncRNAs with prognostic value. **(C)** Survival status and expressive heatmap of 4 m^6^A-LPMs in the training group (red indicates high expression, green indicates low expression). **(D)** Survival status and expressive heatmap of 4 m^6^A-LPMs in the test group (red indicates high expression, green indicates low expression). **(E,F)** Kaplan-Meier curve of 4 m^6^A-LPMs in the training group **(E)** and test group **(F)**. **(G,H)** time-ROC curves of 4 m^6^A-LPMs in the training group **(G)** and test group **(H)**. LASSO, the least absolute shrinkage and selection operator; ROC, the receiver operating characteristic curve; m^6^A-LPMs, m^6^A-related lncRNA prognostic markers. Statistical methods: Chi-square test, log-rank test.

First, univariate Cox regression analysis showed that the differential expression of the six m^6^A-related lncRNAs were closely associated with OS in LUSC patients. Next, the six lncRNAs were performed LASSO regression analysis, and we finally obtained four lncRNAs (AC138035.1, HORMAD2-AS1, AC243919.2 and AL122125.1) and coefficients of each of the lncRNAs to identify the most potent prognostic m^6^A-LPMs (Figures 7A,B). We randomly divided 495 LUSC patients into the training group (248) and the test group (247) in a 1:1 ratio. The clinical characteristics of LUSC patients in the training and test groups were shown in Table 2. Based on the expression and regression coefficients of the 4 m^6^A-LPMs in the training and test groups, we constructed a risk model and calculated the risk score of each patient. Subsequently, all patients were divided into the high-risk group and the low-risk group according to the median risk score. The risk score curve and survival status and the expression heatmap of 4 m^6^A-LPMs in different groups were shown in Figures 7C,D. These results revealed that the survival period of patients gradually decreased, and the number of deaths gradually increased with the increase of the risk score in the training group and the test group (green dots represented live patients, red dots represented dead patients). The heatmap results showed that the HORMAD2-AS1 was highly expressed in the high-risk group compared with the low-risk group, whereas the expression of AC138035.1, AC243919.2, and AL122125.1 was downregulated in the high-risk group compared with the low-risk group (Figures 7C,D). In the LUSC training and test groups, the OS of the low-risk group was significantly longer than the high-risk group (Figures 7E,F). ROC curves was used to assess the prognostic accuracy of this risk model. The area under the curve (AUC) values for 1-year of this risk model in the training and the test groups were 0.617 and 0.572, respectively (Figures 7G,H). These results indicate that this risk model can better predict the prognosis of LUSC patients.

**TABLE 2:** Clinicopathological characteristics of LUSC patients in training and test group.

Covariates	Type	Total	Test	Train	*P* value
Age	≤65	189 (38.18%)	98 (39.68%)	91 (36.69%)	0.6021
	>65	300 (60.61%)	147 (59.51%)	153 (61.69%)	
	unknow	6 (1.21%)	2 (0.81%)	4 (1.61%)	
Gender	FEMALE	129 (26.06%)	68 (27.53%)	61 (24.6%)	0.5215
	MALE	366 (73.94%)	179 (72.47%)	187 (75.4%)	
Stage	Stage I	242 (48.89%)	116 (46.96%)	126 (50.81%)	0.8386
	Stage II	159 (32.12%)	80 (32.39%)	79 (31.85%)	
	Stage III	83 (16.77%)	44 (17.81%)	39 (15.73%)	
	Stage IV	7 (1.41%)	4 (1.62%)	3 (1.21%)	
	unknow	4 (0.81%)	3 (1.21%)	1 (0.4%)	
T	T1	114 (23.03%)	51 (20.65%)	63 (25.4%)	0.288
	T2	288 (58.18%)	142 (57.49%)	146 (58.87%)	
	T3	70 (14.14%)	41 (16.6%)	29 (11.69%)	
	T4	23 (4.65%)	13 (5.26%)	10 (4.03%)	
M	M0	407 (82.22%)	202 (81.78%)	205 (82.66%)	0.8694
	M1	7 (1.41%)	4 (1.62%)	3 (1.21%)	
	MX	77 (15.56%)	40 (16.19%)	37 (14.92%)	
	unknow	4 (0.81%)	1 (0.4%)	3 (1.21%)	
N	N0	316 (63.84%)	158 (63.97%)	158 (63.71%)	0.5429
	N1	128 (25.86%)	62 (25.1%)	66 (26.61%)	
	N2	40 (8.08%)	19 (7.69%)	21 (8.47%)	
	N3	5 (1.01%)	3 (1.21%)	2 (0.81%)	
	NX	6 (1.21%)	5 (2.02%)	1 (0.4%)	

LUSC, lung squamous cell carcinoma; T, T stage; M, M stage; N, N stage.

### 3.7 Correlation Analysis of m^6^A-LPMs With Clinicopathological Features and Immune Score

**FIGURE 8:**
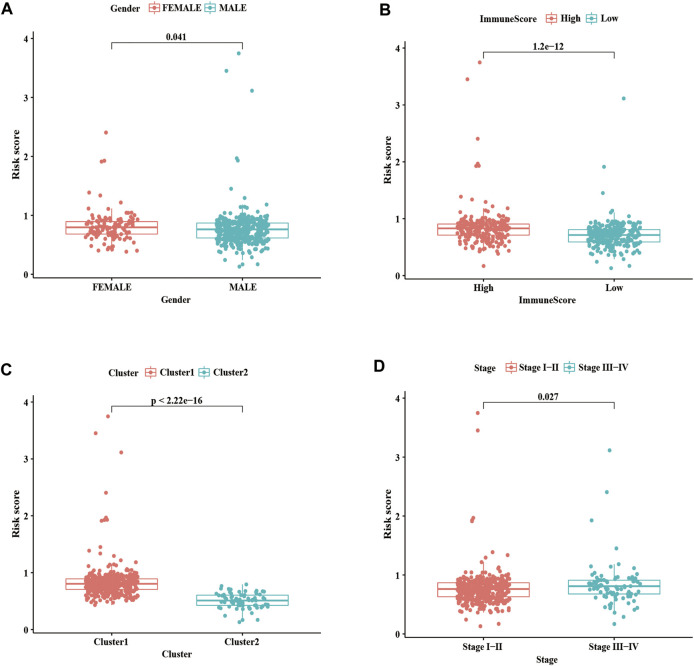
Correlation analysis of risk score with clinicopathological characteristics and immune scores. **(A)** Boxplots of risk score with different genders. **(B)** Boxplots of risk scores and different immune scores. **(C)** Boxplots of risk scores and different clusters. **(D)** Boxplots of risk scores and different Stages. Statistical methods: Chi-square test. ***p < 0.001.

We further evaluated the relationship between the risk score, clinicopathological features, and immune score. As shown in Figure 8A, the risk score in the females is higher than in males; we also found that compared with the high immune score group and cluster 1, the low immune score group and cluster 2 had a lower risk score (Figures 8B,C). As well as, patients with LUSC stage I-II had lower risk scores than those with stage III-IV (Figure 8D). These results showed that the risk score was correlated with TME and tumor malignant stage.

**FIGURE 9:**
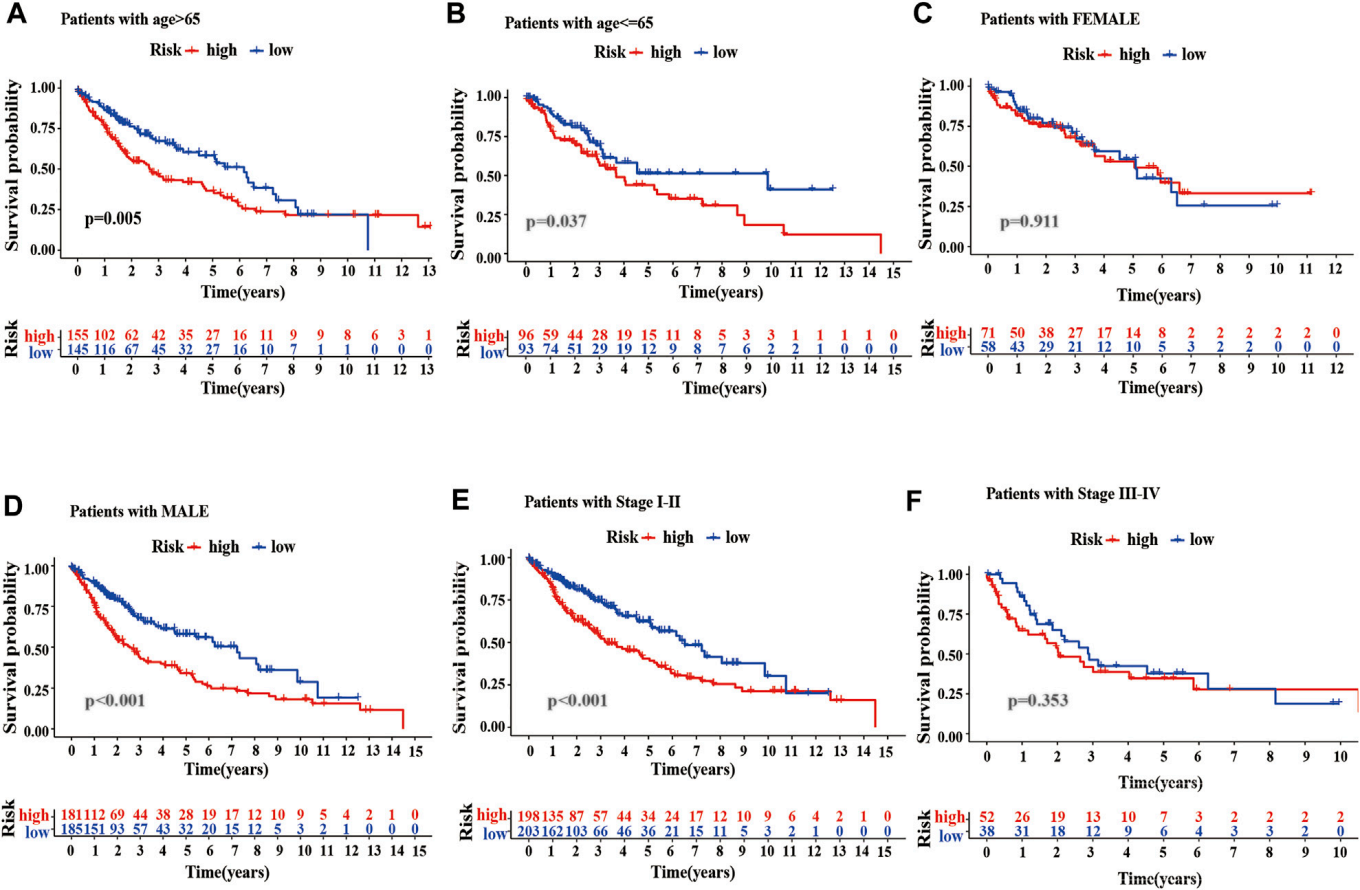
Stratified analysis of survival based on clinicopathological features. **(A–F)** The predictive ability of m^6^A-LPMs in multiple subgroups of LUSC patients, including the different ages: >65 **(A)** or≤65 **(B)**, different sexes: female **(C)** or male **(D)**, different stages: I-II **(E)** or III-IV **(F)**. LUSC, lung squamous cell carcinoma; m^6^A-LPMs, m^6^A-related lncRNA prognostic markers. Statistical methods: Log-rank test.

To better evaluate the prognostic value of m^6^A-LPMs, we further assessed whether they retained the ability to predict OS of patients in different subgroups by stratified analysis. Our analysis found that high-risk patients had poor OS than low-risk patients in all age groups (Figures 9A,B). Gender influenced the ability of m^6^A-LPMs to predict OS in LUSC patients. As is shown in Figures 9C,D, males in the low-risk group had better OS than those in the high-risk group, whereas risk scores did not affect OS in females. In addition, m^6^A-LPMs also predicts OS in LUSC patients with stage I-II but does not predict OS in LUSC patients with stage III-IV patients (Figures 9E,F). Overall, these results indicate that m^6^A-LPMs may be potential predictors for patients with LUSC.

### 3.8 Independent Prognostic Factors for Patients With LUSC

**FIGURE 10:**
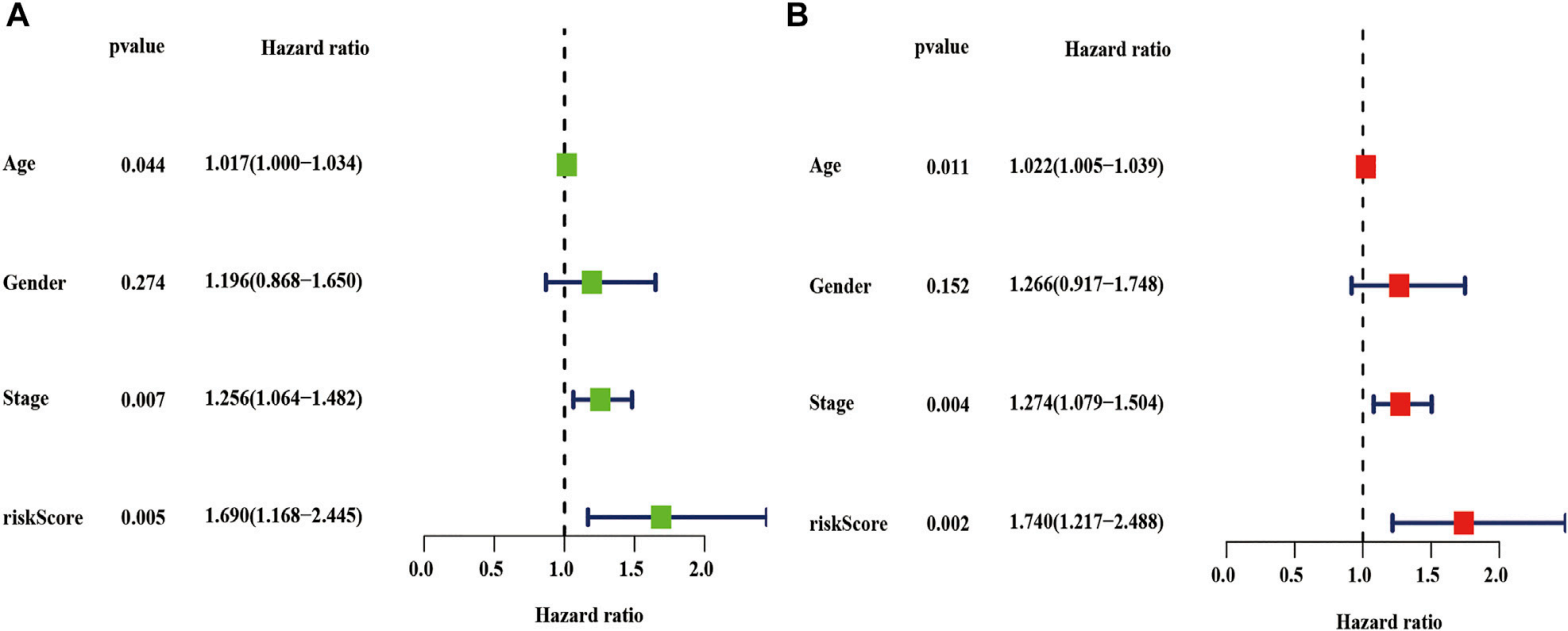
Univariate and multivariate cox analyses of the risk score. **(A–B)** Univariate **(A)** and multivariate **(B)** Cox analyses of the risk score in the TCGA entire dataset. TCGA, the cancer genome atlas.

We also performed univariate and multivariate cox analyses to determine whether the risk score are independent prognostic factors for LUSC patients. As is shown in Figure 10A, univariate Cox analysis showed that the risk score, age, and stage as optimal prognostic factors for LUSC patients in the entire TCGA dataset (the hazard ratio (HR) of risk score was 1.690 and 95% confidence interval (CI) was 1.168–2.445, *p* = 0.005; the HR of age was 1.017 and 95% CI was 1.000–1.034, *p* = 0.044; and the HR of the stage was 1.256 and 95% CI was 1.064–1.482, *p* = 0.007, Supplementary Table S4). Moreover, after adjusting for age, sex, and stage, we found that risk scores was independent prognostic factors for LUSC patients in the TCGA entire set (Figure 10B, Supplementary Table S5).

### 3.9 Correlation Analysis of m^6^A-LPMS in LUSC With Immune Cells

**FIGURE 11:**
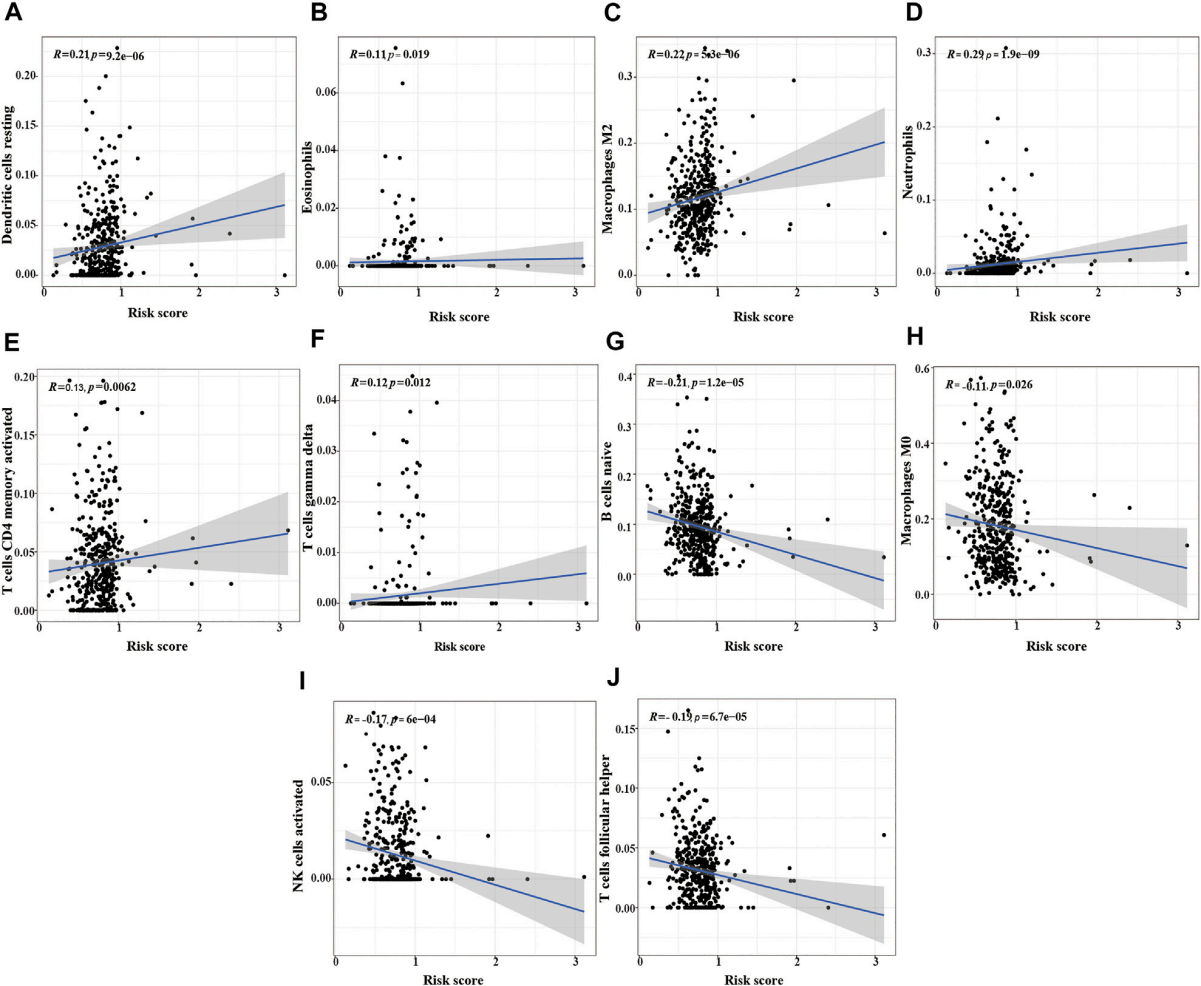
The correlation analysis between the risk score and immune cells. **(A)** resting dendritic cells, **(B)** eosinophils, **(C)** M2 macrophages, **(D)** neutrophils, **(E)** activated CD4 memory activated cells, **(F)** gamma delta T cells, **(G)** naïve B cells, **(H)** M0 macrophages, **(I)** activated NK cells, **(J)** follicular helper T cells.

We also analyzed the relationship between the risk score and immune cells to evaluate the impact of m^6^A-LPMs on the TME of LUSC. We found that the infiltration level of some immune cells (resting dendritic cells, eosinophils, activated M2 macrophages, neutrophils, activated CD4 memory-activated cells, and gamma delta T cells) in LUSC is positively correlated with the risk score (Figures 11A–F). The infiltration level of other immune cells (naïve B cells, non-activated M0 macrophages, activated NK cells, and follicular helper T cells) is negatively correlated with the risk score (Figures 11G–J). These results suggest that m^6^A-LPMS might influence cancer progression by regulating the level of immune cells in LUSC.

### 3.10 Verifying the Expression of m^6^A-LPMs in 16HBE Cell and HCC1588 Cell

**FIGURE 12:**
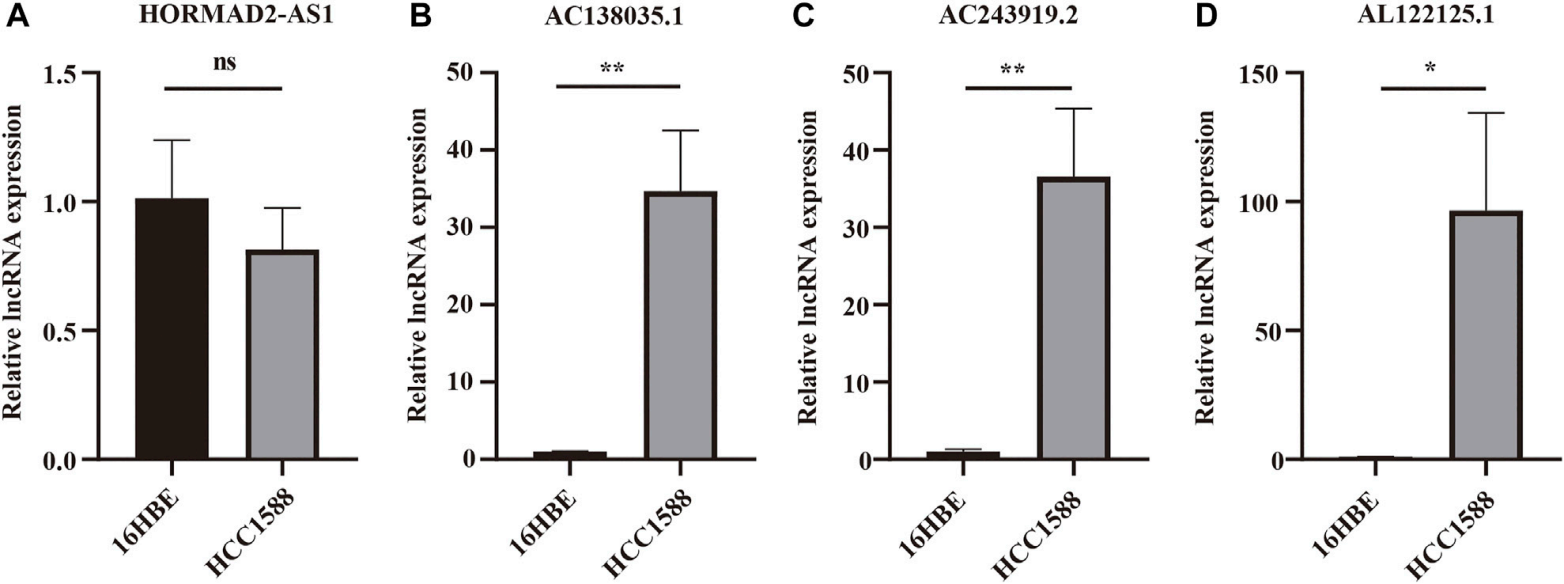
The expression of 4 m^6^A-LPMs in 16HBE cell and HCC1588 cell. **(A–D)** The expression of HORMAD2-AS1 **(A)**, AC138035.1 **(B)**, AC243919.2 **(C)**, and AL122125.1 **(D)** in 16HBE cells and HCC1588 cells were verified by real-time PCR. m^6^A-LPMs, m^6^A-related lncRNA prognostic markers. Statistical methods: *t*-test, *p < 0.05, **p < 0.01.

Real-time PCR was used further to verify the expression of m^6^A-LPMs in different cells. We found that the expression of HORMAD2-AS1 was not statistically significant between the two groups; however, the trend of expression was consistent with our predicted results (Figure 12A). The expression of AC138035.1, AC243919.2, and AL122125.1 in the HCC1588 cell was statistically higher than in the 16HBE cell (Figures 12B–D). We will collect more clinical specimens and cell lines to verify our results.

## 4 Discussion

LUSC has a high rate of metastasis and recurrence. The therapy for LUSC is limited, so that the prognosis of LUSC patients is still poor (Qian et al., 2016). Different subtypes of lung cancer patients have different clinical characteristics and clinical outcomes; therefore, many researchers tend to study non-coding RNA signatures to predict the prognosis and the response to immunotherapy of NSCLC patients (Song et al., 2019; Tian et al., 2020). Some studies have found that m^6^A modification plays an essential role in tumor progression. In several tumors, m^6^A modulators can promote tumor growth and metastasis by regulating the expression of the corresponding lncRNAs. Zheng et al. (2019) found that m^6^A methylation in nasopharyngeal carcinoma reduces the rate of RNA degradation and increases the stability of methylated FAM225A transcripts; it combines with miR-590–3p and miR-1275 to regulate the expression of ITGB3 and activate the FAK/PI3K/AKT pathway to promote the proliferation and invasion of nasopharyngeal carcinoma. Wu et al. (2019) also found that m^6^A-induced lncRNA RP11-triggered epithelial-mesenchymal transition (EMT) by the upregulation of ZEB1 promotes invasion of colorectal cancer cells. Recently, a few studies found that m^6^A regulation of lncRNAs can promote the progression and resistance of NSCLC. For instance, Jin et al. (2019) found that m^6^A mRNA methylation initiated by the m^6^A transferase METL3 promotes YAP mRNA translation by recruiting YTHDF1/3 and iodine trifluoride to the translation initiation complex and increases expression of YAP mRNA by regulating the MALAT1-miR-1914-3p-YAP axis stability. The increase in YAP mRNA expression and activity induces resistance and metastasis of NSCLC. These studies have confirmed that the modification of lncRNAs by m^6^A may affect the occurrence and progression of various cancers, including NSCLC, and that lncRNAs can also target m^6^A regulators to promote tumor progression. However, there have only been a few studies on the mechanism by which m^6^A regulates lncRNAs in LUSC to promote tumor progression. Therefore, more research focused on the interaction between m^6^A modification and lncRNAs is needed to identify potential new targets for the prognosis and treatment of LUSC.

Our study confirmed the expression network of m^6^A-related lncRNAs in LUSC, its prognostic value, its impact on the TME, and its relationship with clinicopathological characteristics. We identified 6 m^6^A-related prognostic lncRNAs from 481 LUSC patients. The expression of 5 lncRNAs (AC138035.1, AP001469.3, AC243919.2, PRC1−AS1, and AL122125.1) in tumor tissues were higher than those in normal adjacent tissues, but the expression levels of lncRNAs HORMAD2−AS1 were lower than those in normal adjacent tissues. Among them, the high expression of AC138035.1 is closely related to the poor prognosis of ovarian cancer patients (Lin H. et al., 2021). Zhao et al. (2021). found that a natural plant peptide aldehyde inhibitor MG-132 (carbobenzoxy-Leu-Leu-leucinal) to inhibit human pterygium fibroblasts (HPFs) proliferate and increase apoptosis by regulating the expression of lncRNAs such as AL122125.1. In addition, we also discovered other lncRNAs for the first time. According to the consensus cluster analysis of the prognostic-related lncRNAs, we identified two subtypes of LUSC, namely cluster 1 and cluster 2. It was found that the expression of PD-L1 in LUSC tumor tissues was lower than that in normal tissues, and the expression of PD-L1 in cluster 2 in LUSC was lower than that in cluster 1. PD-L1 protein expression level has been used as a biomarker to predict which patients are more likely to respond to immunotherapy; and studies have found that the expression level of PD-L1 in NSCLC patients is 24%–60%, which may result from differences in patients’ treatments methods, detection antibodies or demographics (Yu et al., 2016). We found that the immune score and matrix score of cluster 2 in LUSC were lower than cluster 1. However, the immune and matrix scores were not significantly correlated with clinicopathological characteristics and prognosis. Our findings are similar to some previous studies. For example, Qu et al. (2020) found that the matrix score had no significant correlation with clinical characteristics and prognosis for LUSC cases. In cluster 1 of LUSC, there were higher infiltration levels of resting CD4 memory T cells, activated M2 macrophages, neutrophils, regulatory T cells (Tregs), and resting NK cells than in cluster 2. However, the infiltration levels of naïve B cells, follicular helper T cells, and activated NK cells were higher in cluster 2 than in cluster 1. The results of the GSEA showed that focal adhesion, ECM receptor interaction, and cytokine receptors related to the malignant characteristics of tumors were mainly found in cluster 1. Focal adhesion is a non-receptor tyrosine kinase involved in cancer cell growth, proliferation, survival, migration, angiogenesis, invasion, and EMT, essential for maintaining cancer stem cells and macrophages (Soria et al., 2016). m^6^A modification directly controls RNA metabolism, including mRNA processing, output, translation initiation and maintenance stability, and the biogenesis of lncRNA, which may be involved in the regulation of cytokines, immune response, and the self-renewal ability of cancer stem cells and play a key role in cell proliferation and apoptosis (Chang et al., 2019). Therefore, we suggest that the differences in TME between different subgroups of LUSC are regulated by the FAK pathway and cytokine receptors.

Subsequently, we evaluated and verified the prognostic value of 4 m^6^A-related-LPMs in LUSC patients. Compared with bronchial epithelial cells, the expression of AC138035.1, AC243919.2, and AL122125.1 was increased in HCC1588 cells by real-time PCR, while the expression of HORMAD2−AS1 was not statistically significant. The limitation of cells number, lacking verified tests by tissues, and tumor specificity may be the main reasons. More clinical specimens and cell lines should be used to enhance the research credibility by further experimental proof as the study of these lncRNAs remains unknown. We divide LUSC patients into high- and low-risk groups by risk score. In the LUSC training group and test group, the OS of low-risk patients was longer than that of high-risk patients. In addition, the AUC values for 1 year OS were 0.617 and 0.572 in the training and test groups, respectively. These results indicate that this risk model can better predict the prognosis of LUSC patients. Previous studies have shown that the root mean square difference between the estimated and true metrics is significant in the case of a small sample size, which may affect the real performance of the ROC curve and lead to a low AUC value (Hanczar et al., 2010; Berrar and Flach, 2012) Since our data come from the TCGA database, the possible reason for the low AUC value is related to the small sample size in our different groups. The risk score of the male, low immune score group, and cluster 2 was lower than those of the female, high immune score group, and cluster 1. The stratified analysis revealed that the prognostic characteristics of m^6^A-LPMs retained the ability to predict the OS rate of different ages, male patients, and early tumor patients. We also confirmed that the risk score could be an independent prognostic factor for LUSC patients by univariate and multivariate Cox regression analysis. Previous studies have confirmed that the increased expression level of AC138035.1 is closely related to the poor prognosis of patients with ovarian cancer (Lin N. et al., 2021). These studies show that m^6^A-LPMs may be effective in predicting the prognosis of LUSC patients.

The TME plays a vital role in the development of tumors and affects the treatment and prognosis of patients (Fridman et al., 2012; Galon et al., 2014; Hui and Chen, 2015). However, the mechanism of the interaction between immune infiltration and tumor in LUSC remains unclear, and the understanding of m^6^A-modified lncRNAs in the regulation of TME is still limited. Previous studies have confirmed a large number of neutrophil infiltration in the high-grade invasive histological subtypes of LUAD, which is closely related to the poor prognosis of LUAD patients (Kurebayashi et al., 2016). Neutrophils exist in the tumor immune microenvironment of the tumor and can boost tumor progression by promoting cell growth, angiogenesis, metastasis, and immune evasion (Mollaoglu et al., 2018). Macrophages are classified into M1 and M2 macrophages according to their functions. Studies have found that the M1 macrophages exert pro-inflammatory effects and anti-tumor immunity, while M2 macrophages participate in anti-inflammatory response and promote tumor progression effects (Chanmee et al., 2014). Some studies have found that increased eosinophils in tumor tissues can improve the prognosis of patients, but eosinophils are related to poor prognosis in Hodgkin lymphoma (Davis and Rothenberg, 2014). Our study discovered that the infiltration level of some immune cells (resting dendritic cells, eosinophils, activated M2 macrophages, neutrophils, activated CD4 memory T cells, and gamma delta T cells) is positively correlated with the risk score and poor prognosis of LUSC patients. At the same time, we also found that other immune cells (naïve B cells, follicular helper T cells, activated NK cells, non-activated M0 macrophages) infiltration level is negatively correlated with a risk score. Chen et al., 2020b have found that naïve-like B cells not only inhibit the proliferation of NSCLC cells but are also associated with a good prognosis of NSCLC; the expression of NK cells in tumor tissues is positively correlated with the prognosis of cancer patients. NK cells can enhance the ability of antibodies and T cells to recognize tumors, and activated NK cells can eliminate the cancer cells in the circulating blood and overcome tumor resistance (Shimasaki and Jain, 2020). Infiltrating follicular helper T cells play a protective role in breast and colon cancer, and its increased expression level in tumors indicates that it has a better prognosis for patients (Bindea et al., 2013; Gu-Trantien et al., 2013). The above results indicated that m^6^A modified lncRNAs might affect patients with LUSC by regulating TME. Therefore, it is urgent to study further the regulatory role of m^6^A-related lncRNAs in the TME of LUSC.

However, there are still some defects in our research. Firstly, our data mainly comes from the lung cancer cohort in the TCGA database, and more external validation is needed in the future to assess whether it can be used in clinical patients. Secondly, because of the limited number of cells and tumor tissues, there might be a slight deviation in the validation of m^6^A-LPMs; moreover, the molecular mechanism and the role of these lncRNAs in tumor growth, metastasis, and immune infiltration need to be further studied. Therefore, in subsequent studies, we will further verify our results by experiments *in vivo* and *in vitro*; and investigate the impact of m^6^A and its prognostic-related lncRNAs on the TME to improve the efficacy of immunotherapy LUSC patients.

## 5 Conclusion

In conclusion, we found that 4 m^6^A-LPMs (AC138035.1, AC243919.2, HORMAD2-AS1 and AL122125.1) are closely associated with LUSC patients’ prognoses. We also divided LUSC patients into high-risk and low-risk groups according to the risk score of the m^6^A-related LPMs. This risk score was found to be closely related to the immune score, clinicopathological characteristics, and the level of immune cell infiltration of LUSC patients, and it was also found to be an independent prognostic indicator of LUSC patients. These biomarkers are beneficial to the treatment and prognosis of LUSC patients. Further study of their potential regulatory mechanisms may identify novel targets for the prognosis and immunotherapy of LUSC patients.

## Data Availability

The datasets generated and analyzed during this study are available in the TCGA database (https://portal.gdc.cancer.gov). The original contributions presented in the study are included in the Article/Supplementary Material; further inquiries can be directed to the corresponding authors.
