# Supplementary material for: N 6 -Methyladenosine-Related Long Non-Coding RNAs Are Identified as a Potential Prognostic Biomarker for Lung Squamous Cell Carcinoma and Validated by Real-Time PCR
Source: Front Genet. 2022 Jun 3;13:839957. doi: 10.3389/fgene.2022.839957 (PMC9204524; doi:10.3389/fgene.2022.839957)
Supplement: Supplementary file 1 [file Table1.DOCX]

**Table S1** m^6^A regulatory genes and corresponding lncRNA in LUSC

| m6A | lncRNA | cor | pvalue | Regulation |
| --- | --- | --- | --- | --- |
| METTL3 | AC087392.3 | 0.422621441 | 3.63E-23 | postive |
| WTAP | AC068790.8 | 0.434361164 | 1.62E-24 | postive |
| METTL3 | AC245060.2 | 0.455921944 | 3.89E-27 | postive |
| YTHDC2 | AC245060.2 | 0.407470354 | 1.68E-21 | postive |
| METTL3 | AC087741.1 | 0.49062798 | 9.12E-32 | postive |
| IGFBP1 | AL354696.1 | 0.43054118 | 4.52E-24 | postive |
| ZC3H13 | AC073046.1 | 0.456212197 | 3.58E-27 | postive |
| HNRNPA2B1 | AP001505.1 | 0.457340532 | 2.58E-27 | postive |
| YTHDF2 | AL391244.2 | 0.406364505 | 2.21E-21 | postive |
| YTHDC2 | AL050343.2 | 0.445014506 | 8.69E-26 | postive |
| METTL3 | AP000873.4 | 0.440384343 | 3.14E-25 | postive |
| YTHDC2 | AL031600.1 | 0.411565559 | 6.07E-22 | postive |
| METTL3 | STAM-AS1 | 0.407473703 | 1.68E-21 | postive |
| HNRNPA2B1 | STAM-AS1 | 0.409569731 | 9.99E-22 | postive |
| METTL3 | AC138393.3 | 0.415186356 | 2.44E-22 | postive |
| IGFBP2 | AC010980.2 | 0.418806547 | 9.71E-23 | postive |
| YTHDC2 | AL390208.1 | 0.42973754 | 5.60E-24 | postive |
| METTL3 | AL160314.2 | 0.433173112 | 2.23E-24 | postive |
| RBMX | AP001347.1 | 0.409789613 | 9.46E-22 | postive |
| HNRNPA2B1 | AL442128.2 | 0.429355507 | 6.20E-24 | postive |
| METTL3 | AF111169.3 | 0.408719851 | 1.23E-21 | postive |
| METTL3 | AC232271.1 | 0.460864799 | 9.17E-28 | postive |
| YTHDC1 | AC232271.1 | 0.414024709 | 3.27E-22 | postive |
| YTHDC2 | AC232271.1 | 0.422190328 | 4.06E-23 | postive |
| HNRNPA2B1 | AC232271.1 | 0.451266476 | 1.48E-26 | postive |
| YTHDF1 | AL096828.3 | 0.445320409 | 7.97E-26 | postive |
| HNRNPA2B1 | AC025176.1 | 0.407326382 | 1.74E-21 | postive |
| YTHDC2 | AL158163.1 | 0.493566201 | 3.49E-32 | postive |
| IGFBP1 | AC060780.1 | 0.70578705 | 7.07E-77 | postive |
| YTHDC1 | AC037459.2 | 0.409711512 | 9.64E-22 | postive |
| YTHDC2 | AC037459.2 | 0.415334996 | 2.35E-22 | postive |
| YTHDC2 | PRR7-AS1 | 0.40212664 | 6.20E-21 | postive |
| METTL3 | AC027601.2 | 0.53641352 | 9.56E-39 | postive |
| YTHDC2 | AC027601.2 | 0.41749813 | 1.36E-22 | postive |
| YTHDC2 | NFYC-AS1 | 0.410585946 | 7.76E-22 | postive |
| METTL3 | NCBP2-AS1 | 0.455850098 | 3.97E-27 | postive |
| YTHDF2 | MRPL20-AS1 | 0.407250801 | 1.77E-21 | postive |
| METTL3 | AL391684.1 | 0.477097657 | 6.73E-30 | postive |
| YTHDC2 | AL391684.1 | 0.402628294 | 5.49E-21 | postive |
| ZC3H13 | AC234775.3 | 0.411884182 | 5.61E-22 | postive |
| RBMX | AL592435.1 | 0.459087174 | 1.55E-27 | postive |
| METTL3 | AL136295.6 | 0.462980866 | 4.91E-28 | postive |
| HNRNPC | SNHG30 | 0.480087135 | 2.65E-30 | postive |
| METTL3 | AL022328.3 | 0.449229808 | 2.65E-26 | postive |
| YTHDC2 | AL022328.3 | 0.412901291 | 4.35E-22 | postive |
| METTL3 | AC013731.1 | 0.417948627 | 1.21E-22 | postive |
| YTHDC2 | AC116366.2 | 0.469616392 | 6.70E-29 | postive |
| METTL3 | AC005104.1 | 0.422630967 | 3.62E-23 | postive |
| YTHDC2 | AC005104.1 | 0.402350638 | 5.88E-21 | postive |
| RBMX | AC012531.1 | 0.446554199 | 5.64E-26 | postive |
| YTHDC1 | AC112484.1 | 0.420934184 | 5.62E-23 | postive |
| METTL3 | AP002907.1 | 0.467968578 | 1.10E-28 | postive |
| YTHDC1 | AC138035.1 | 0.402794891 | 5.27E-21 | postive |
| HNRNPA2B1 | AC138035.1 | 0.473110612 | 2.31E-29 | postive |
| METTL3 | AC004908.2 | 0.415442945 | 2.29E-22 | postive |
| METTL3 | AL121852.1 | 0.408188777 | 1.41E-21 | postive |
| METTL3 | AC144548.1 | 0.424962394 | 1.97E-23 | postive |
| YTHDC1 | AC144548.1 | 0.479779448 | 2.91E-30 | postive |
| YTHDC2 | AC144548.1 | 0.40337378 | 4.58E-21 | postive |
| HNRNPA2B1 | AC144548.1 | 0.423748584 | 2.71E-23 | postive |
| RBMX | LINC01397 | 0.469694971 | 6.54E-29 | postive |
| METTL3 | AC008764.6 | 0.404856463 | 3.19E-21 | postive |
| METTL3 | PTOV1-AS2 | 0.459107243 | 1.54E-27 | postive |
| YTHDC1 | PTOV1-AS2 | 0.408866792 | 1.19E-21 | postive |
| YTHDC2 | PTOV1-AS2 | 0.461369966 | 7.90E-28 | postive |
| HNRNPA2B1 | PTOV1-AS2 | 0.460368083 | 1.06E-27 | postive |
| METTL3 | AC008434.1 | 0.448646476 | 3.13E-26 | postive |
| YTHDC2 | AC008434.1 | 0.429436008 | 6.07E-24 | postive |
| IGFBP1 | HORMAD2-AS1 | 0.43531864 | 1.25E-24 | postive |
| RBMX | MIR924HG | 0.508655855 | 2.17E-34 | postive |
| METTL3 | SNHG21 | 0.400587979 | 9.00E-21 | postive |
| METTL3 | AC093249.2 | 0.409928188 | 9.14E-22 | postive |
| RBMX | AC006449.2 | 0.409573155 | 9.98E-22 | postive |
| YTHDC2 | AL049795.1 | 0.433780773 | 1.90E-24 | postive |
| IGFBP1 | LINC01996 | 0.95224585 | 9.12E-260 | postive |
| METTL3 | AC084125.2 | 0.47550759 | 1.10E-29 | postive |
| METTL3 | AC132872.2 | 0.411831891 | 5.68E-22 | postive |
| METTL3 | AC020911.1 | 0.422930757 | 3.35E-23 | postive |
| HNRNPA2B1 | AC078909.2 | 0.423773585 | 2.69E-23 | postive |
| METTL3 | SPAG5-AS1 | 0.465871724 | 2.07E-28 | postive |
| YTHDC1 | SPAG5-AS1 | 0.416109038 | 1.93E-22 | postive |
| YTHDC2 | SPAG5-AS1 | 0.486063812 | 3.98E-31 | postive |
| HNRNPA2B1 | SPAG5-AS1 | 0.468797969 | 8.58E-29 | postive |
| RBMX | CRTC3-AS1 | 0.491373116 | 7.15E-32 | postive |
| METTL3 | PSMA3-AS1 | 0.551340119 | 2.92E-41 | postive |
| YTHDC1 | PSMA3-AS1 | 0.436067739 | 1.02E-24 | postive |
| YTHDC2 | PSMA3-AS1 | 0.42359058 | 2.82E-23 | postive |
| HNRNPA2B1 | C1RL-AS1 | 0.411139465 | 6.76E-22 | postive |
| YTHDC2 | SLC25A25-AS1 | 0.438883867 | 4.74E-25 | postive |
| IGFBP1 | LINC01767 | 0.415967126 | 2.00E-22 | postive |
| METTL3 | AL161668.3 | 0.653413099 | 1.92E-62 | postive |
| RBMX | CACNA1C-AS1 | 0.400348429 | 9.53E-21 | postive |
| METTL3 | AC137630.2 | 0.400784098 | 8.58E-21 | postive |
| METTL3 | SNHG20 | 0.530389504 | 9.13E-38 | postive |
| HNRNPA2B1 | SNHG20 | 0.412500631 | 4.81E-22 | postive |
| YTHDC2 | AC103691.1 | 0.410400585 | 8.12E-22 | postive |
| HNRNPA2B1 | AC103691.1 | 0.404813688 | 3.23E-21 | postive |
| HNRNPA2B1 | AP001469.3 | 0.421850152 | 4.43E-23 | postive |
| METTL3 | AC073575.4 | 0.425854047 | 1.56E-23 | postive |
| METTL3 | AC145423.3 | 0.420504723 | 6.28E-23 | postive |
| METTL3 | AC020558.2 | 0.507818834 | 2.90E-34 | postive |
| HNRNPA2B1 | AC020558.2 | 0.417513625 | 1.35E-22 | postive |
| METTL3 | AL451050.2 | 0.422060218 | 4.20E-23 | postive |
| RBMX | AC008115.3 | 0.40761897 | 1.62E-21 | postive |
| METTL3 | AC253536.6 | 0.413597327 | 3.65E-22 | postive |
| METTL3 | AP006621.2 | 0.468580773 | 9.17E-29 | postive |
| HNRNPA2B1 | AC010719.1 | 0.418642983 | 1.01E-22 | postive |
| METTL3 | AL132989.2 | 0.423305618 | 3.04E-23 | postive |
| HNRNPA2B1 | AC079174.2 | 0.438923244 | 4.69E-25 | postive |
| METTL3 | AL359921.1 | 0.409097526 | 1.12E-21 | postive |
| METTL3 | AC022167.2 | 0.425114869 | 1.90E-23 | postive |
| HNRNPA2B1 | SMG7-AS1 | 0.401851223 | 6.63E-21 | postive |
| METTL16 | COX10-AS1 | 0.444501813 | 1.00E-25 | postive |
| YTHDC2 | AC093495.1 | 0.422747476 | 3.51E-23 | postive |
| YTHDC2 | AC007406.5 | 0.409959642 | 9.07E-22 | postive |
| METTL3 | AC087294.1 | 0.405232789 | 2.91E-21 | postive |
| METTL3 | AL356019.2 | 0.402642617 | 5.47E-21 | postive |
| YTHDC2 | AP000442.1 | 0.400210441 | 9.85E-21 | postive |
| METTL3 | AC025766.1 | 0.447059175 | 4.89E-26 | postive |
| YTHDC2 | AC025766.1 | 0.434614662 | 1.52E-24 | postive |
| METTL3 | AC022211.2 | 0.432105979 | 2.98E-24 | postive |
| METTL3 | AC006435.2 | 0.500352662 | 3.67E-33 | postive |
| HNRNPA2B1 | AC006557.1 | 0.481095478 | 1.93E-30 | postive |
| METTL3 | AC007038.1 | 0.422996416 | 3.29E-23 | postive |
| HNRNPA2B1 | AC007038.1 | 0.43173629 | 3.29E-24 | postive |
| YTHDC2 | AC005674.2 | 0.413930638 | 3.35E-22 | postive |
| METTL3 | AL031705.1 | 0.411085221 | 6.85E-22 | postive |
| METTL3 | AL592211.1 | 0.451090859 | 1.56E-26 | postive |
| HNRNPA2B1 | AL592211.1 | 0.425632532 | 1.65E-23 | postive |
| FMR1 | FMR1-IT1 | 0.534784641 | 1.77E-38 | postive |
| METTL3 | AC104564.3 | 0.435121042 | 1.32E-24 | postive |
| YTHDC2 | AC104564.3 | 0.44988815 | 2.20E-26 | postive |
| IGFBP1 | AL117382.2 | 0.666299613 | 1.01E-65 | postive |
| YTHDC2 | AC116914.2 | 0.413512508 | 3.73E-22 | postive |
| HNRNPA2B1 | AC092171.4 | 0.402087171 | 6.26E-21 | postive |
| METTL3 | DHDDS-AS1 | 0.428974298 | 6.86E-24 | postive |
| YTHDC2 | DHDDS-AS1 | 0.408679473 | 1.25E-21 | postive |
| HNRNPA2B1 | DHDDS-AS1 | 0.413941862 | 3.34E-22 | postive |
| METTL3 | AC004148.1 | 0.509600986 | 1.57E-34 | postive |
| YTHDC2 | AC004148.1 | 0.462580869 | 5.52E-28 | postive |
| HNRNPA2B1 | AC004148.1 | 0.43757649 | 6.78E-25 | postive |
| METTL3 | AC007292.1 | 0.423752217 | 2.71E-23 | postive |
| HNRNPA2B1 | AC007292.1 | 0.468384574 | 9.73E-29 | postive |
| METTL3 | AC008764.8 | 0.485735952 | 4.42E-31 | postive |
| YTHDC2 | AC008764.8 | 0.426024435 | 1.49E-23 | postive |
| HNRNPA2B1 | AC008764.8 | 0.437434358 | 7.05E-25 | postive |
| HNRNPA2B1 | AC125494.1 | 0.459862262 | 1.23E-27 | postive |
| METTL3 | AL590729.1 | 0.414864315 | 2.65E-22 | postive |
| HNRNPA2B1 | AL590729.1 | 0.422093195 | 4.16E-23 | postive |
| METTL3 | AC087289.2 | 0.450435017 | 1.88E-26 | postive |
| HNRNPA2B1 | AC087289.2 | 0.40112906 | 7.90E-21 | postive |
| IGFBP1 | LINC01943 | 0.559866433 | 9.34E-43 | postive |
| HNRNPA2B1 | AC017083.1 | 0.426451144 | 1.33E-23 | postive |
| METTL3 | AC245060.6 | 0.414134191 | 3.19E-22 | postive |
| METTL3 | AC010618.2 | 0.445491138 | 7.60E-26 | postive |
| IGFBP1 | LINC00892 | 0.487414364 | 2.58E-31 | postive |
| METTL3 | AC010973.2 | 0.442960301 | 1.54E-25 | postive |
| YTHDC2 | AP003486.1 | 0.411683467 | 5.90E-22 | postive |
| YTHDC2 | LINC00641 | 0.400185151 | 9.91E-21 | postive |
| METTL3 | RUSC1-AS1 | 0.481181759 | 1.87E-30 | postive |
| HNRNPA2B1 | RUSC1-AS1 | 0.46090621 | 9.06E-28 | postive |
| IGFBP1 | B3GALT1-AS1 | 0.921415377 | 2.12E-207 | postive |
| YTHDC2 | AC093726.2 | 0.401096453 | 7.96E-21 | postive |
| METTL16 | AC027763.2 | 0.400166204 | 9.96E-21 | postive |
| METTL3 | AC005519.1 | 0.545287199 | 3.16E-40 | postive |
| METTL3 | AP001628.1 | 0.401052373 | 8.04E-21 | postive |
| HNRNPA2B1 | AP001628.1 | 0.423964065 | 2.56E-23 | postive |
| HNRNPA2B1 | TMPO-AS1 | 0.443745773 | 1.24E-25 | postive |
| RBMX | TMPO-AS1 | 0.521723208 | 2.17E-36 | postive |
| RBMX | AL358472.2 | 0.432584162 | 2.62E-24 | postive |
| METTL3 | AC106782.5 | 0.469434336 | 7.08E-29 | postive |
| METTL3 | RNF139-AS1 | 0.54079594 | 1.80E-39 | postive |
| HNRNPA2B1 | RNF139-AS1 | 0.442022469 | 2.00E-25 | postive |
| METTL3 | AC018766.1 | 0.400618719 | 8.93E-21 | postive |
| YTHDC2 | AC018766.1 | 0.412790513 | 4.47E-22 | postive |
| YTHDC2 | AC015871.3 | 0.473786324 | 1.87E-29 | postive |
| RBMX | LINC01138 | 0.44325755 | 1.42E-25 | postive |
| YTHDC1 | AC012360.3 | 0.432193114 | 2.91E-24 | postive |
| YTHDC2 | AC012360.3 | 0.412577581 | 4.71E-22 | postive |
| HNRNPA2B1 | AC012360.3 | 0.498882299 | 6.00E-33 | postive |
| METTL3 | AC023355.2 | 0.462420642 | 5.79E-28 | postive |
| HNRNPA2B1 | AC023355.2 | 0.448741853 | 3.04E-26 | postive |
| METTL3 | LINC01424 | 0.433928698 | 1.82E-24 | postive |
| HNRNPA2B1 | LINC01424 | 0.435894712 | 1.07E-24 | postive |
| IGFBP1 | AC083900.1 | 0.64987475 | 1.43E-61 | postive |
| METTL3 | TMEM147-AS1 | 0.49873544 | 6.31E-33 | postive |
| HNRNPA2B1 | TMEM147-AS1 | 0.405496243 | 2.73E-21 | postive |
| YTHDC2 | AC104532.2 | 0.487578078 | 2.45E-31 | postive |
| METTL3 | AL390719.2 | 0.409264189 | 1.08E-21 | postive |
| METTL3 | AL021707.6 | 0.43564613 | 1.15E-24 | postive |
| YTHDC2 | AL021707.6 | 0.420062822 | 7.03E-23 | postive |
| HNRNPA2B1 | AL021707.6 | 0.465055183 | 2.64E-28 | postive |
| METTL3 | LINC01355 | 0.471473104 | 3.81E-29 | postive |
| YTHDC1 | LINC01355 | 0.423742104 | 2.71E-23 | postive |
| HNRNPA2B1 | LINC01355 | 0.455746563 | 4.09E-27 | postive |
| YTHDC2 | AC008906.1 | 0.499947529 | 4.20E-33 | postive |
| METTL3 | GUSBP11 | 0.430184583 | 4.97E-24 | postive |
| METTL3 | AC005306.1 | 0.449908448 | 2.19E-26 | postive |
| YTHDC2 | AC005306.1 | 0.432619904 | 2.59E-24 | postive |
| METTL3 | AP000553.2 | 0.414213594 | 3.12E-22 | postive |
| METTL3 | AC004477.1 | 0.401854754 | 6.63E-21 | postive |
| YTHDC2 | NPTN-IT1 | 0.40578723 | 2.54E-21 | postive |
| METTL3 | AC055855.2 | 0.411557 | 6.09E-22 | postive |
| YTHDC2 | AC055855.2 | 0.429385489 | 6.15E-24 | postive |
| HNRNPA2B1 | AC055855.2 | 0.421213117 | 5.23E-23 | postive |
| METTL3 | AC079907.1 | 0.479560942 | 3.12E-30 | postive |
| HNRNPA2B1 | AC079907.1 | 0.420904013 | 5.66E-23 | postive |
| METTL3 | AL928654.2 | 0.552241038 | 2.04E-41 | postive |
| METTL3 | AL356299.3 | 0.420633972 | 6.07E-23 | postive |
| METTL3 | AL139353.2 | 0.46953037 | 6.88E-29 | postive |
| METTL3 | AC010319.4 | 0.411972885 | 5.48E-22 | postive |
| HNRNPA2B1 | AC010319.4 | 0.40695838 | 1.91E-21 | postive |
| METTL14 | AL606807.1 | 0.447727316 | 4.05E-26 | postive |
| METTL3 | AC092611.1 | 0.406706865 | 2.03E-21 | postive |
| RBMX | AC097534.1 | 0.482648147 | 1.18E-30 | postive |
| METTL3 | AC009148.1 | 0.441583573 | 2.26E-25 | postive |
| HNRNPA2B1 | AC009148.1 | 0.410752435 | 7.44E-22 | postive |
| HNRNPA2B1 | AC002398.1 | 0.43263001 | 2.59E-24 | postive |
| HNRNPA2B1 | AC127024.5 | 0.474616629 | 1.45E-29 | postive |
| RBMX | AC127024.5 | 0.40528538 | 2.87E-21 | postive |
| YTHDC2 | AC008735.2 | 0.436335608 | 9.50E-25 | postive |
| METTL3 | ZNF32-AS2 | 0.424253709 | 2.37E-23 | postive |
| YTHDC1 | ZNF32-AS2 | 0.427990461 | 8.90E-24 | postive |
| YTHDC2 | ZNF32-AS2 | 0.440098003 | 3.40E-25 | postive |
| HNRNPA2B1 | ZNF32-AS2 | 0.409962634 | 9.06E-22 | postive |
| HNRNPA2B1 | AC106820.3 | 0.466444895 | 1.74E-28 | postive |
| METTL3 | LINC00115 | 0.448590368 | 3.18E-26 | postive |
| METTL3 | AC027601.3 | 0.496487601 | 1.33E-32 | postive |
| METTL3 | AC006480.3 | 0.432885459 | 2.41E-24 | postive |
| YTHDC2 | AC006480.3 | 0.437520003 | 6.89E-25 | postive |
| METTL3 | AC022973.5 | 0.400555467 | 9.07E-21 | postive |
| METTL3 | AC011472.1 | 0.450758258 | 1.72E-26 | postive |
| ZC3H13 | AC108449.2 | 0.426545874 | 1.30E-23 | postive |
| HNRNPA2B1 | SNHG1 | 0.421746584 | 4.55E-23 | postive |
| RBMX | SNHG1 | 0.418327748 | 1.10E-22 | postive |
| YTHDC2 | AC091887.1 | 0.43894358 | 4.67E-25 | postive |
| HNRNPA2B1 | AC078778.1 | 0.413537094 | 3.70E-22 | postive |
| METTL3 | AC105137.2 | 0.436912667 | 8.12E-25 | postive |
| IGFBP2 | DIRC3 | 0.427342943 | 1.06E-23 | postive |
| YTHDC2 | MANEA-DT | 0.469423117 | 7.10E-29 | postive |
| METTL3 | AP006621.3 | 0.416361707 | 1.81E-22 | postive |
| YTHDC1 | AC243919.2 | 0.437890952 | 6.22E-25 | postive |
| YTHDC1 | AC120053.1 | 0.467541818 | 1.25E-28 | postive |
| HNRNPA2B1 | AC120053.1 | 0.502942311 | 1.53E-33 | postive |
| IGFBP1 | AC104461.1 | 0.51825076 | 7.53E-36 | postive |
| METTL3 | AL021707.8 | 0.447323027 | 4.54E-26 | postive |
| YTHDC2 | AL021707.8 | 0.444874203 | 9.03E-26 | postive |
| METTL3 | AL132780.2 | 0.444818468 | 9.18E-26 | postive |
| METTL3 | TMED2-DT | 0.442269032 | 1.87E-25 | postive |
| HNRNPA2B1 | ASB16-AS1 | 0.43899023 | 4.61E-25 | postive |
| IGFBP3 | AC023669.2 | 0.430071027 | 5.12E-24 | postive |
| METTL3 | AC016737.1 | 0.401269661 | 7.63E-21 | postive |
| METTL3 | SEMA3F-AS1 | 0.403186082 | 4.80E-21 | postive |
| YTHDC2 | SEMA3F-AS1 | 0.445446299 | 7.70E-26 | postive |
| METTL3 | ZKSCAN2-DT | 0.458366113 | 1.91E-27 | postive |
| YTHDC1 | ZKSCAN2-DT | 0.435425494 | 1.22E-24 | postive |
| YTHDC2 | ZKSCAN2-DT | 0.423072359 | 3.23E-23 | postive |
| HNRNPA2B1 | ZKSCAN2-DT | 0.473670151 | 1.94E-29 | postive |
| YTHDC2 | AC015802.4 | 0.403376393 | 4.58E-21 | postive |
| FMR1 | AL683813.2 | 0.508376025 | 2.39E-34 | postive |
| RBMX | AL683813.2 | 0.437409526 | 7.10E-25 | postive |
| YTHDC2 | AC109587.1 | 0.48630099 | 3.69E-31 | postive |
| METTL3 | AC012615.6 | 0.455637758 | 4.22E-27 | postive |
| YTHDC2 | AC012615.6 | 0.427121948 | 1.12E-23 | postive |
| METTL3 | AC016773.2 | 0.432866943 | 2.43E-24 | postive |
| HNRNPA2B1 | AC016773.2 | 0.400441415 | 9.32E-21 | postive |
| METTL3 | AL139099.3 | 0.425339282 | 1.79E-23 | postive |
| YTHDC2 | AL450263.1 | 0.436505398 | 9.08E-25 | postive |
| METTL3 | AL132780.1 | 0.651091837 | 7.21E-62 | postive |
| METTL3 | ZFHX2-AS1 | 0.681433161 | 8.67E-70 | postive |
| WTAP | CCNT2-AS1 | 0.43919427 | 4.36E-25 | postive |
| METTL3 | AP001029.1 | 0.424653708 | 2.14E-23 | postive |
| METTL3 | AP003352.1 | 0.455418285 | 4.50E-27 | postive |
| METTL3 | SNHG10 | 0.498257035 | 7.40E-33 | postive |
| METTL3 | SNHG12 | 0.400728896 | 8.70E-21 | postive |
| YTHDC2 | AC093788.1 | 0.4242293 | 2.39E-23 | postive |
| HNRNPA2B1 | AC093788.1 | 0.422673277 | 3.58E-23 | postive |
| HNRNPA2B1 | AL121906.1 | 0.42279366 | 3.47E-23 | postive |
| YTHDF2 | AC098484.1 | 0.42958028 | 5.84E-24 | postive |
| IGFBP1 | ADORA2A-AS1 | 0.459482052 | 1.38E-27 | postive |
| METTL3 | ZNF436-AS1 | 0.405881588 | 2.48E-21 | postive |
| YTHDC1 | ZNF436-AS1 | 0.436534752 | 9.00E-25 | postive |
| HNRNPA2B1 | ZNF436-AS1 | 0.407836853 | 1.53E-21 | postive |
| METTL3 | AC091057.1 | 0.416860661 | 1.60E-22 | postive |
| RBM15 | AC091057.1 | 0.442031117 | 1.99E-25 | postive |
| YTHDC1 | AC091057.1 | 0.473150817 | 2.28E-29 | postive |
| HNRNPA2B1 | AC091057.1 | 0.580747742 | 1.32E-46 | postive |
| WTAP | MAPKAPK5-AS1 | 0.4510556 | 1.58E-26 | postive |
| HNRNPC | MAPKAPK5-AS1 | 0.509214935 | 1.79E-34 | postive |
| RBMX | MAPKAPK5-AS1 | 0.403280367 | 4.69E-21 | postive |
| METTL3 | AL096701.3 | 0.429594195 | 5.82E-24 | postive |
| METTL3 | AC127024.4 | 0.41627076 | 1.85E-22 | postive |
| YTHDC2 | AC127024.4 | 0.408351077 | 1.35E-21 | postive |
| METTL3 | SCAT2 | 0.480261228 | 2.50E-30 | postive |
| HNRNPA2B1 | SCAT2 | 0.47031526 | 5.42E-29 | postive |
| METTL3 | AC092910.3 | 0.414746099 | 2.73E-22 | postive |
| METTL3 | AC087289.1 | 0.411145177 | 6.75E-22 | postive |
| HNRNPA2B1 | AC087289.1 | 0.433322924 | 2.15E-24 | postive |
| YTHDC2 | AC090948.2 | 0.412388285 | 4.94E-22 | postive |
| IGFBP1 | AC080038.3 | 0.480331743 | 2.45E-30 | postive |
| HNRNPA2B1 | AC004951.1 | 0.416845166 | 1.60E-22 | postive |
| METTL3 | AC023908.3 | 0.449409758 | 2.52E-26 | postive |
| YTHDC2 | AC023908.3 | 0.43444727 | 1.59E-24 | postive |
| HNRNPA2B1 | AC023908.3 | 0.422909871 | 3.37E-23 | postive |
| METTL3 | AC137932.3 | 0.412460629 | 4.85E-22 | postive |
| HNRNPA2B1 | AC137932.3 | 0.40549456 | 2.73E-21 | postive |
| METTL3 | AC139530.1 | 0.449182614 | 2.69E-26 | postive |
| METTL3 | AC074117.1 | 0.451801654 | 1.27E-26 | postive |
| HNRNPA2B1 | AC074117.1 | 0.436141347 | 1.00E-24 | postive |
| METTL3 | AL096870.2 | 0.630460765 | 5.55E-57 | postive |
| METTL3 | AP001001.1 | 0.431792444 | 3.24E-24 | postive |
| RBMX | ARNILA | 0.402621471 | 5.50E-21 | postive |
| RBMX | STX18-AS1 | 0.425706319 | 1.62E-23 | postive |
| METTL3 | AP001107.4 | 0.431943599 | 3.11E-24 | postive |
| ZC3H13 | AP000766.1 | 0.405456074 | 2.76E-21 | postive |
| YTHDC2 | AP000766.1 | 0.415694863 | 2.15E-22 | postive |
| YTHDF3 | YTHDF3-AS1 | 0.432901259 | 2.40E-24 | postive |
| IGFBP1 | AC008915.3 | 0.575902686 | 1.09E-45 | postive |
| YTHDC2 | AC136604.2 | 0.40938133 | 1.05E-21 | postive |
| METTL3 | AP001160.1 | 0.50265776 | 1.69E-33 | postive |
| METTL3 | AC022558.3 | 0.462522954 | 5.62E-28 | postive |
| YTHDC1 | AC022558.3 | 0.426750093 | 1.23E-23 | postive |
| YTHDC2 | AC022558.3 | 0.449893126 | 2.20E-26 | postive |
| HNRNPA2B1 | AC022558.3 | 0.45600172 | 3.80E-27 | postive |
| METTL3 | AC245052.4 | 0.401383239 | 7.43E-21 | postive |
| HNRNPA2B1 | AC245052.4 | 0.451986522 | 1.21E-26 | postive |
| METTL3 | AL354989.1 | 0.405173294 | 2.95E-21 | postive |
| METTL3 | AC010976.1 | 0.452739917 | 9.74E-27 | postive |
| YTHDC2 | AC010976.1 | 0.418912233 | 9.45E-23 | postive |
| METTL3 | AL132639.3 | 0.496500715 | 1.33E-32 | postive |
| METTL3 | AC114730.3 | 0.488002 | 2.13E-31 | postive |
| YTHDC1 | MIR17HG | 0.41452452 | 2.89E-22 | postive |
| HNRNPA2B1 | MIR17HG | 0.411797268 | 5.73E-22 | postive |
| METTL3 | AL135999.1 | 0.674062649 | 8.89E-68 | postive |
| YTHDC2 | AL135999.1 | 0.400513118 | 9.16E-21 | postive |
| METTL3 | LINC01089 | 0.490471247 | 9.59E-32 | postive |
| METTL3 | LINC01311 | 0.404199158 | 3.75E-21 | postive |
| YTHDC2 | AC010245.2 | 0.455523563 | 4.37E-27 | postive |
| METTL3 | AC233728.1 | 0.402662051 | 5.45E-21 | postive |
| HNRNPA2B1 | PRC1-AS1 | 0.41425106 | 3.09E-22 | postive |
| METTL3 | AC022211.4 | 0.469278796 | 7.42E-29 | postive |
| HNRNPA2B1 | AC002550.2 | 0.406019346 | 2.40E-21 | postive |
| HNRNPA2B1 | LINC00205 | 0.435735707 | 1.12E-24 | postive |
| YTHDC1 | AC015813.1 | 0.413873515 | 3.40E-22 | postive |
| METTL3 | AL121832.3 | 0.445599549 | 7.38E-26 | postive |
| YTHDC1 | AL121832.3 | 0.411905681 | 5.58E-22 | postive |
| HNRNPA2B1 | AL121832.3 | 0.451398234 | 1.43E-26 | postive |
| METTL3 | LINC00894 | 0.457656834 | 2.35E-27 | postive |
| YTHDC2 | LINC00894 | 0.440097919 | 3.40E-25 | postive |
| YTHDC2 | AC084876.1 | 0.430043148 | 5.16E-24 | postive |
| FMR1 | AC245140.2 | 0.42007161 | 7.02E-23 | postive |
| METTL3 | AL161756.1 | 0.528613702 | 1.76E-37 | postive |
| METTL3 | AL031186.1 | 0.451187168 | 1.52E-26 | postive |
| HNRNPA2B1 | AL031186.1 | 0.435180983 | 1.30E-24 | postive |
| METTL3 | AL139287.1 | 0.464296888 | 3.32E-28 | postive |
| YTHDC1 | AL139287.1 | 0.458845435 | 1.66E-27 | postive |
| HNRNPA2B1 | AL139287.1 | 0.451537652 | 1.37E-26 | postive |
| IGFBP1 | AC007036.1 | 0.420057807 | 7.04E-23 | postive |
| METTL3 | AL122125.1 | 0.424462483 | 2.25E-23 | postive |
| IGFBP1 | AL162411.1 | 0.774192823 | 2.23E-101 | postive |
| YTHDC2 | SH3BP5-AS1 | 0.486045359 | 4.00E-31 | postive |
| YTHDC1 | AC007878.1 | 0.405012387 | 3.07E-21 | postive |
| RBMX | AL354892.2 | 0.453288509 | 8.32E-27 | postive |
| YTHDC1 | AL157392.3 | 0.440313283 | 3.20E-25 | postive |
| YTHDC2 | AL157392.3 | 0.431865023 | 3.17E-24 | postive |
| METTL3 | MORF4L2-AS1 | 0.458337999 | 1.93E-27 | postive |
| IGFBP1 | LINC00513 | 0.539811691 | 2.62E-39 | postive |
| RBMX | LINC00526 | 0.403661396 | 4.27E-21 | postive |
| METTL3 | AC109460.3 | 0.457960059 | 2.15E-27 | postive |
| IGFBP2 | AC073321.1 | 0.519123529 | 5.51E-36 | postive |
| HNRNPA2B1 | AC069222.1 | 0.415598043 | 2.20E-22 | postive |
| RBMX | ZBED3-AS1 | 0.43982425 | 3.66E-25 | postive |
| HNRNPA2B1 | AL662797.2 | 0.496205575 | 1.46E-32 | postive |
| METTL3 | UBE2Q1-AS1 | 0.523746971 | 1.04E-36 | postive |
| YTHDC2 | UBE2Q1-AS1 | 0.414571013 | 2.85E-22 | postive |
| METTL3 | AC092119.2 | 0.443382912 | 1.37E-25 | postive |
| YTHDC2 | AC092119.2 | 0.406892134 | 1.94E-21 | postive |
| METTL3 | GTF3C2-AS1 | 0.455718326 | 4.13E-27 | postive |
| HNRNPA2B1 | GTF3C2-AS1 | 0.424283703 | 2.35E-23 | postive |
| YTHDC1 | THAP9-AS1 | 0.511912417 | 7.03E-35 | postive |
| METTL3 | AL139123.1 | 0.495654901 | 1.75E-32 | postive |
| HNRNPA2B1 | AL139123.1 | 0.400561629 | 9.05E-21 | postive |
| HNRNPC | UBL7-AS1 | 0.408609793 | 1.27E-21 | postive |
| ZC3H13 | AC010834.3 | 0.422297657 | 3.95E-23 | postive |
| YTHDC1 | AC010834.3 | 0.508196355 | 2.55E-34 | postive |
| HNRNPA2B1 | AC010834.3 | 0.462356783 | 5.90E-28 | postive |
| HNRNPC | PXN-AS1 | 0.413093468 | 4.14E-22 | postive |
| RBMX | ID2-AS1 | 0.404794877 | 3.24E-21 | postive |
| METTL3 | AL133243.3 | 0.421239497 | 5.19E-23 | postive |
| METTL3 | AP001107.1 | 0.444266455 | 1.07E-25 | postive |
| RBMX | TRAF3IP2-AS1 | 0.448898002 | 2.91E-26 | postive |
| HNRNPA2B1 | AC010422.2 | 0.446868269 | 5.16E-26 | postive |
| METTL3 | AL138921.1 | 0.423604883 | 2.81E-23 | postive |
| METTL3 | AC018809.1 | 0.457289354 | 2.61E-27 | postive |
| METTL3 | AC008870.2 | 0.449212893 | 2.66E-26 | postive |
| YTHDC2 | AC008870.2 | 0.441232942 | 2.48E-25 | postive |
| HNRNPA2B1 | AC008870.2 | 0.438773173 | 4.89E-25 | postive |
| METTL3 | AC009065.9 | 0.428731756 | 7.31E-24 | postive |
| HNRNPA2B1 | AC009065.9 | 0.427298625 | 1.07E-23 | postive |
| HNRNPA2B1 | AC020663.3 | 0.405142411 | 2.98E-21 | postive |
| METTL3 | AC002128.1 | 0.446334158 | 6.00E-26 | postive |
| YTHDC2 | AC066613.1 | 0.411444606 | 6.26E-22 | postive |
| YTHDC2 | AP000254.2 | 0.427917212 | 9.07E-24 | postive |
| HNRNPA2B1 | AP000254.2 | 0.408138494 | 1.42E-21 | postive |
| METTL3 | ERVK13-1 | 0.408785918 | 1.21E-21 | postive |
| YTHDC2 | ERVK13-1 | 0.43727548 | 7.36E-25 | postive |
| YTHDC2 | ARHGAP27P1-BPTFP1-KPNA2P3 | 0.402141602 | 6.18E-21 | postive |
| METTL3 | AC005785.1 | 0.44546431 | 7.66E-26 | postive |
| METTL3 | AC010761.1 | 0.493915847 | 3.11E-32 | postive |
| HNRNPA2B1 | AC010761.1 | 0.435329972 | 1.25E-24 | postive |
| METTL3 | AC022098.1 | 0.423249524 | 3.08E-23 | postive |
| METTL3 | AL354733.3 | 0.459681214 | 1.30E-27 | postive |
| YTHDC2 | AL354733.3 | 0.455410557 | 4.51E-27 | postive |
| YTHDC2 | MIR3936HG | 0.450530246 | 1.83E-26 | postive |
| METTL3 | MCM3AP-AS1 | 0.424009544 | 2.53E-23 | postive |
| YTHDC1 | MCM3AP-AS1 | 0.408285746 | 1.37E-21 | postive |
| HNRNPA2B1 | MCM3AP-AS1 | 0.424891456 | 2.01E-23 | postive |
| METTL3 | AL161452.1 | 0.446017308 | 6.56E-26 | postive |
| HNRNPA2B1 | AL161452.1 | 0.428666604 | 7.44E-24 | postive |
| YTHDC2 | FTX | 0.40144198 | 7.32E-21 | postive |
| YTHDC2 | PSMD6-AS2 | 0.444035918 | 1.14E-25 | postive |
| METTL3 | AC084018.1 | 0.488300507 | 1.94E-31 | postive |
| YTHDC2 | AC084018.1 | 0.43121391 | 3.78E-24 | postive |
| METTL3 | ZNF252P-AS1 | 0.508292121 | 2.47E-34 | postive |
| HNRNPA2B1 | ZNF252P-AS1 | 0.411091721 | 6.84E-22 | postive |
| METTL3 | CAPN10-DT | 0.475580351 | 1.08E-29 | postive |
| HNRNPA2B1 | CAPN10-DT | 0.436932206 | 8.08E-25 | postive |
| METTL3 | LINC02878 | 0.449982856 | 2.14E-26 | postive |
| IGFBP1 | AC023511.1 | 0.547700062 | 1.23E-40 | postive |
| METTL14 | AC027702.2 | 0.400887204 | 8.37E-21 | postive |
| IGFBP1 | LINC01871 | 0.650150014 | 1.23E-61 | postive |
| METTL3 | INTS6-AS1 | 0.424593456 | 2.17E-23 | postive |
| METTL3 | AC132192.1 | 0.408276773 | 1.38E-21 | postive |
| METTL3 | AL355488.1 | 0.444340479 | 1.05E-25 | postive |
| YTHDC2 | AL355488.1 | 0.47000282 | 5.96E-29 | postive |
| HNRNPA2B1 | AL355488.1 | 0.488760089 | 1.67E-31 | postive |
| METTL3 | AP001793.1 | 0.426044388 | 1.49E-23 | postive |
| YTHDC2 | AP001793.1 | 0.417059202 | 1.52E-22 | postive |
| METTL3 | AL132989.1 | 0.456419008 | 3.37E-27 | postive |
| YTHDC2 | AL132989.1 | 0.436358557 | 9.45E-25 | postive |
| YTHDC1 | AL139286.1 | 0.41577646 | 2.10E-22 | postive |
| HNRNPA2B1 | AL139286.1 | 0.422494252 | 3.75E-23 | postive |
| METTL3 | AC010168.2 | 0.439407781 | 4.11E-25 | postive |
| RBM15 | AC012073.1 | 0.429180573 | 6.49E-24 | postive |
| HNRNPA2B1 | AC012073.1 | 0.492064842 | 5.71E-32 | postive |
| METTL3 | AC132192.2 | 0.418266939 | 1.11E-22 | postive |
| HNRNPA2B1 | AC132192.2 | 0.409850148 | 9.32E-22 | postive |
| YTHDC2 | AC092301.1 | 0.434193171 | 1.70E-24 | postive |
| HNRNPA2B1 | AC092301.1 | 0.422741186 | 3.52E-23 | postive |
| METTL3 | AC007390.1 | 0.543069711 | 7.49E-40 | postive |
| YTHDC1 | AC007390.1 | 0.417171467 | 1.47E-22 | postive |
| HNRNPA2B1 | AC007390.1 | 0.470391414 | 5.29E-29 | postive |
| IGFBP1 | AC090809.1 | 0.481448057 | 1.72E-30 | postive |
| METTL3 | AL355388.1 | 0.487348361 | 2.63E-31 | postive |
| METTL3 | AC109460.2 | 0.539357894 | 3.12E-39 | postive |
| YTHDC1 | AC109460.2 | 0.451204451 | 1.51E-26 | postive |
| YTHDC2 | AC109460.2 | 0.431854079 | 3.18E-24 | postive |
| HNRNPA2B1 | AC109460.2 | 0.448393389 | 3.36E-26 | postive |
| IGFBP1 | LINC02195 | 0.70837007 | 1.13E-77 | postive |
| METTL3 | PRMT5-AS1 | 0.636168929 | 2.69E-58 | postive |
| METTL3 | AL022328.2 | 0.467914498 | 1.12E-28 | postive |
| YTHDC2 | AL022328.2 | 0.411389652 | 6.35E-22 | postive |
| METTL3 | AL049780.1 | 0.533731792 | 2.62E-38 | postive |
| YTHDC1 | AL603832.1 | 0.413386561 | 3.85E-22 | postive |
| METTL14 | LINC01402 | 0.431239233 | 3.75E-24 | postive |
| YTHDC2 | AC090198.1 | 0.418015017 | 1.19E-22 | postive |
| YTHDC2 | AC087500.1 | 0.428056107 | 8.74E-24 | postive |
| METTL3 | C1orf220 | 0.43742335 | 7.07E-25 | postive |
| METTL3 | AC005253.1 | 0.45358452 | 7.64E-27 | postive |
| METTL3 | AC078846.1 | 0.413640064 | 3.61E-22 | postive |
| YTHDC2 | RBM5-AS1 | 0.435742892 | 1.12E-24 | postive |
| METTL3 | AC009022.1 | 0.447891608 | 3.87E-26 | postive |
| HNRNPC | SNHG29 | 0.437333254 | 7.24E-25 | postive |
| METTL3 | AC068831.2 | 0.413689052 | 3.56E-22 | postive |
| METTL3 | AC245060.5 | 0.439855287 | 3.63E-25 | postive |
| YTHDC2 | AC245060.5 | 0.420379221 | 6.48E-23 | postive |
| HNRNPA2B1 | AC245060.5 | 0.436922603 | 8.10E-25 | postive |
| METTL3 | STAG3L5P-PVRIG2P-PILRB | 0.401817242 | 6.69E-21 | postive |
| YTHDC2 | STAG3L5P-PVRIG2P-PILRB | 0.414220006 | 3.12E-22 | postive |
| HNRNPA2B1 | STAG3L5P-PVRIG2P-PILRB | 0.445163087 | 8.33E-26 | postive |
| YTHDC2 | SEMA6A-AS1 | 0.507975657 | 2.75E-34 | postive |
| METTL3 | AC084018.2 | 0.421156434 | 5.30E-23 | postive |
| IGFBP1 | LINC00539 | 0.556858489 | 3.18E-42 | postive |
| YTHDC2 | AL031846.2 | 0.427947124 | 9.00E-24 | postive |
| IGFBP1 | AC083841.1 | 0.429360209 | 6.19E-24 | postive |
| METTL3 | AC009095.1 | 0.448678134 | 3.10E-26 | postive |
| YTHDC1 | AC009095.1 | 0.42163468 | 4.69E-23 | postive |
| RBM15 | AL160006.1 | 0.442682641 | 1.66E-25 | postive |
| METTL3 | AC024361.3 | 0.458649134 | 1.76E-27 | postive |
| METTL3 | ZNRD2-AS1 | 0.431711669 | 3.31E-24 | postive |
| YTHDC1 | AP001462.1 | 0.424464412 | 2.25E-23 | postive |
| METTL3 | AC009690.2 | 0.460057097 | 1.16E-27 | postive |
| RBM15B | MIR4435-2HG | -0.416339804 | 1.82E-22 | negative |

m^6^A, *N^6^*-methyladenosine; lncRNA, long non-coding RNA; cor, correlation coefficient
