# Supplementary material for: N 6 -Methyladenosine-Related Long Non-Coding RNAs Are Identified as a Potential Prognostic Biomarker for Lung Squamous Cell Carcinoma and Validated by Real-Time PCR
Source: Front Genet. 2022 Jun 3;13:839957. doi: 10.3389/fgene.2022.839957 (PMC9204524; doi:10.3389/fgene.2022.839957)
Supplement: Supplementary file 2 [file Table2.DOCX]

**Table S2** m^6^A-related lncRNAs in LUSC patients

| Node | Type |
| --- | --- |
| AC087392.3 | lncRNA |
| AC068790.8 | lncRNA |
| AC245060.2 | lncRNA |
| AC087741.1 | lncRNA |
| AL354696.1 | lncRNA |
| AC073046.1 | lncRNA |
| AP001505.1 | lncRNA |
| AL391244.2 | lncRNA |
| AL050343.2 | lncRNA |
| AP000873.4 | lncRNA |
| AL031600.1 | lncRNA |
| STAM-AS1 | lncRNA |
| AC138393.3 | lncRNA |
| AC010980.2 | lncRNA |
| AL390208.1 | lncRNA |
| AL160314.2 | lncRNA |
| AP001347.1 | lncRNA |
| AL442128.2 | lncRNA |
| AF111169.3 | lncRNA |
| AC232271.1 | lncRNA |
| AL096828.3 | lncRNA |
| AC025176.1 | lncRNA |
| AL158163.1 | lncRNA |
| AC060780.1 | lncRNA |
| AC037459.2 | lncRNA |
| PRR7-AS1 | lncRNA |
| AC027601.2 | lncRNA |
| NFYC-AS1 | lncRNA |
| NCBP2-AS1 | lncRNA |
| MRPL20-AS1 | lncRNA |
| AL391684.1 | lncRNA |
| AC234775.3 | lncRNA |
| AL592435.1 | lncRNA |
| AL136295.6 | lncRNA |
| SNHG30 | lncRNA |
| AL022328.3 | lncRNA |
| AC013731.1 | lncRNA |
| AC116366.2 | lncRNA |
| AC005104.1 | lncRNA |
| AC012531.1 | lncRNA |
| AC112484.1 | lncRNA |
| AP002907.1 | lncRNA |
| AC138035.1 | lncRNA |
| AC004908.2 | lncRNA |
| AL121852.1 | lncRNA |
| AC144548.1 | lncRNA |
| LINC01397 | lncRNA |
| AC008764.6 | lncRNA |
| PTOV1-AS2 | lncRNA |
| AC008434.1 | lncRNA |
| HORMAD2-AS1 | lncRNA |
| MIR924HG | lncRNA |
| SNHG21 | lncRNA |
| AC093249.2 | lncRNA |
| AC006449.2 | lncRNA |
| AL049795.1 | lncRNA |
| LINC01996 | lncRNA |
| AC084125.2 | lncRNA |
| AC132872.2 | lncRNA |
| AC020911.1 | lncRNA |
| AC078909.2 | lncRNA |
| SPAG5-AS1 | lncRNA |
| CRTC3-AS1 | lncRNA |
| PSMA3-AS1 | lncRNA |
| C1RL-AS1 | lncRNA |
| SLC25A25-AS1 | lncRNA |
| LINC01767 | lncRNA |
| AL161668.3 | lncRNA |
| CACNA1C-AS1 | lncRNA |
| AC137630.2 | lncRNA |
| SNHG20 | lncRNA |
| AC103691.1 | lncRNA |
| AP001469.3 | lncRNA |
| AC073575.4 | lncRNA |
| AC145423.3 | lncRNA |
| AC020558.2 | lncRNA |
| AL451050.2 | lncRNA |
| AC008115.3 | lncRNA |
| AC253536.6 | lncRNA |
| AP006621.2 | lncRNA |
| AC010719.1 | lncRNA |
| AL132989.2 | lncRNA |
| AC079174.2 | lncRNA |
| AL359921.1 | lncRNA |
| AC022167.2 | lncRNA |
| SMG7-AS1 | lncRNA |
| COX10-AS1 | lncRNA |
| AC093495.1 | lncRNA |
| AC007406.5 | lncRNA |
| AC087294.1 | lncRNA |
| AL356019.2 | lncRNA |
| AP000442.1 | lncRNA |
| AC025766.1 | lncRNA |
| AC022211.2 | lncRNA |
| AC006435.2 | lncRNA |
| AC006557.1 | lncRNA |
| AC007038.1 | lncRNA |
| AC005674.2 | lncRNA |
| AL031705.1 | lncRNA |
| AL592211.1 | lncRNA |
| FMR1-IT1 | lncRNA |
| AC104564.3 | lncRNA |
| AL117382.2 | lncRNA |
| AC116914.2 | lncRNA |
| AC092171.4 | lncRNA |
| DHDDS-AS1 | lncRNA |
| AC004148.1 | lncRNA |
| AC007292.1 | lncRNA |
| AC008764.8 | lncRNA |
| AC125494.1 | lncRNA |
| AL590729.1 | lncRNA |
| AC087289.2 | lncRNA |
| LINC01943 | lncRNA |
| AC017083.1 | lncRNA |
| AC245060.6 | lncRNA |
| AC010618.2 | lncRNA |
| LINC00892 | lncRNA |
| AC010973.2 | lncRNA |
| AP003486.1 | lncRNA |
| LINC00641 | lncRNA |
| RUSC1-AS1 | lncRNA |
| B3GALT1-AS1 | lncRNA |
| AC093726.2 | lncRNA |
| AC027763.2 | lncRNA |
| AC005519.1 | lncRNA |
| AP001628.1 | lncRNA |
| TMPO-AS1 | lncRNA |
| AL358472.2 | lncRNA |
| AC106782.5 | lncRNA |
| RNF139-AS1 | lncRNA |
| AC018766.1 | lncRNA |
| AC015871.3 | lncRNA |
| LINC01138 | lncRNA |
| AC012360.3 | lncRNA |
| AC023355.2 | lncRNA |
| LINC01424 | lncRNA |
| AC083900.1 | lncRNA |
| TMEM147-AS1 | lncRNA |
| AC104532.2 | lncRNA |
| AL390719.2 | lncRNA |
| AL021707.6 | lncRNA |
| LINC01355 | lncRNA |
| AC008906.1 | lncRNA |
| GUSBP11 | lncRNA |
| AC005306.1 | lncRNA |
| AP000553.2 | lncRNA |
| AC004477.1 | lncRNA |
| NPTN-IT1 | lncRNA |
| AC055855.2 | lncRNA |
| AC079907.1 | lncRNA |
| AL928654.2 | lncRNA |
| AL356299.3 | lncRNA |
| AL139353.2 | lncRNA |
| AC010319.4 | lncRNA |
| AL606807.1 | lncRNA |
| AC092611.1 | lncRNA |
| AC097534.1 | lncRNA |
| AC009148.1 | lncRNA |
| AC002398.1 | lncRNA |
| AC127024.5 | lncRNA |
| AC008735.2 | lncRNA |
| ZNF32-AS2 | lncRNA |
| AC106820.3 | lncRNA |
| LINC00115 | lncRNA |
| AC027601.3 | lncRNA |
| AC006480.3 | lncRNA |
| AC022973.5 | lncRNA |
| AC011472.1 | lncRNA |
| AC108449.2 | lncRNA |
| SNHG1 | lncRNA |
| AC091887.1 | lncRNA |
| AC078778.1 | lncRNA |
| AC105137.2 | lncRNA |
| DIRC3 | lncRNA |
| MANEA-DT | lncRNA |
| AP006621.3 | lncRNA |
| AC243919.2 | lncRNA |
| AC120053.1 | lncRNA |
| AC104461.1 | lncRNA |
| AL021707.8 | lncRNA |
| AL132780.2 | lncRNA |
| TMED2-DT | lncRNA |
| ASB16-AS1 | lncRNA |
| AC023669.2 | lncRNA |
| AC016737.1 | lncRNA |
| SEMA3F-AS1 | lncRNA |
| ZKSCAN2-DT | lncRNA |
| AC015802.4 | lncRNA |
| AL683813.2 | lncRNA |
| AC109587.1 | lncRNA |
| AC012615.6 | lncRNA |
| AC016773.2 | lncRNA |
| AL139099.3 | lncRNA |
| AL450263.1 | lncRNA |
| AL132780.1 | lncRNA |
| ZFHX2-AS1 | lncRNA |
| CCNT2-AS1 | lncRNA |
| AP001029.1 | lncRNA |
| AP003352.1 | lncRNA |
| SNHG10 | lncRNA |
| SNHG12 | lncRNA |
| AC093788.1 | lncRNA |
| AL121906.1 | lncRNA |
| AC098484.1 | lncRNA |
| ADORA2A-AS1 | lncRNA |
| ZNF436-AS1 | lncRNA |
| AC091057.1 | lncRNA |
| MAPKAPK5-AS1 | lncRNA |
| AL096701.3 | lncRNA |
| AC127024.4 | lncRNA |
| SCAT2 | lncRNA |
| AC092910.3 | lncRNA |
| AC087289.1 | lncRNA |
| AC090948.2 | lncRNA |
| AC080038.3 | lncRNA |
| AC004951.1 | lncRNA |
| AC023908.3 | lncRNA |
| AC137932.3 | lncRNA |
| AC139530.1 | lncRNA |
| AC074117.1 | lncRNA |
| AL096870.2 | lncRNA |
| AP001001.1 | lncRNA |
| ARNILA | lncRNA |
| STX18-AS1 | lncRNA |
| AP001107.4 | lncRNA |
| AP000766.1 | lncRNA |
| YTHDF3-AS1 | lncRNA |
| AC008915.3 | lncRNA |
| AC136604.2 | lncRNA |
| AP001160.1 | lncRNA |
| AC022558.3 | lncRNA |
| AC245052.4 | lncRNA |
| AL354989.1 | lncRNA |
| AC010976.1 | lncRNA |
| AL132639.3 | lncRNA |
| AC114730.3 | lncRNA |
| MIR17HG | lncRNA |
| AL135999.1 | lncRNA |
| LINC01089 | lncRNA |
| LINC01311 | lncRNA |
| AC010245.2 | lncRNA |
| AC233728.1 | lncRNA |
| PRC1-AS1 | lncRNA |
| AC022211.4 | lncRNA |
| AC002550.2 | lncRNA |
| LINC00205 | lncRNA |
| AC015813.1 | lncRNA |
| AL121832.3 | lncRNA |
| LINC00894 | lncRNA |
| AC084876.1 | lncRNA |
| AC245140.2 | lncRNA |
| AL161756.1 | lncRNA |
| AL031186.1 | lncRNA |
| AL139287.1 | lncRNA |
| AC007036.1 | lncRNA |
| AL122125.1 | lncRNA |
| AL162411.1 | lncRNA |
| SH3BP5-AS1 | lncRNA |
| AC007878.1 | lncRNA |
| AL354892.2 | lncRNA |
| AL157392.3 | lncRNA |
| MORF4L2-AS1 | lncRNA |
| LINC00513 | lncRNA |
| LINC00526 | lncRNA |
| AC109460.3 | lncRNA |
| AC073321.1 | lncRNA |
| AC069222.1 | lncRNA |
| ZBED3-AS1 | lncRNA |
| AL662797.2 | lncRNA |
| UBE2Q1-AS1 | lncRNA |
| AC092119.2 | lncRNA |
| GTF3C2-AS1 | lncRNA |
| THAP9-AS1 | lncRNA |
| AL139123.1 | lncRNA |
| UBL7-AS1 | lncRNA |
| AC010834.3 | lncRNA |
| PXN-AS1 | lncRNA |
| ID2-AS1 | lncRNA |
| AL133243.3 | lncRNA |
| AP001107.1 | lncRNA |
| TRAF3IP2-AS1 | lncRNA |
| AC010422.2 | lncRNA |
| AL138921.1 | lncRNA |
| AC018809.1 | lncRNA |
| AC008870.2 | lncRNA |
| AC009065.9 | lncRNA |
| AC020663.3 | lncRNA |
| AC002128.1 | lncRNA |
| AC066613.1 | lncRNA |
| AP000254.2 | lncRNA |
| ERVK13-1 | lncRNA |
| ARHGAP27P1-BPTFP1-KPNA2P3 | lncRNA |
| AC005785.1 | lncRNA |
| AC010761.1 | lncRNA |
| AC022098.1 | lncRNA |
| AL354733.3 | lncRNA |
| MIR3936HG | lncRNA |
| MCM3AP-AS1 | lncRNA |
| AL161452.1 | lncRNA |
| FTX | lncRNA |
| PSMD6-AS2 | lncRNA |
| AC084018.1 | lncRNA |
| ZNF252P-AS1 | lncRNA |
| CAPN10-DT | lncRNA |
| LINC02878 | lncRNA |
| AC023511.1 | lncRNA |
| AC027702.2 | lncRNA |
| LINC01871 | lncRNA |
| INTS6-AS1 | lncRNA |
| AC132192.1 | lncRNA |
| AL355488.1 | lncRNA |
| AP001793.1 | lncRNA |
| AL132989.1 | lncRNA |
| AL139286.1 | lncRNA |
| AC010168.2 | lncRNA |
| AC012073.1 | lncRNA |
| AC132192.2 | lncRNA |
| AC092301.1 | lncRNA |
| AC007390.1 | lncRNA |
| AC090809.1 | lncRNA |
| AL355388.1 | lncRNA |
| AC109460.2 | lncRNA |
| LINC02195 | lncRNA |
| PRMT5-AS1 | lncRNA |
| AL022328.2 | lncRNA |
| AL049780.1 | lncRNA |
| AL603832.1 | lncRNA |
| LINC01402 | lncRNA |
| AC090198.1 | lncRNA |
| AC087500.1 | lncRNA |
| C1orf220 | lncRNA |
| AC005253.1 | lncRNA |
| AC078846.1 | lncRNA |
| RBM5-AS1 | lncRNA |
| AC009022.1 | lncRNA |
| SNHG29 | lncRNA |
| AC068831.2 | lncRNA |
| AC245060.5 | lncRNA |
| STAG3L5P-PVRIG2P-PILRB | lncRNA |
| SEMA6A-AS1 | lncRNA |
| AC084018.2 | lncRNA |
| LINC00539 | lncRNA |
| AL031846.2 | lncRNA |
| AC083841.1 | lncRNA |
| AC009095.1 | lncRNA |
| AL160006.1 | lncRNA |
| AC024361.3 | lncRNA |
| ZNRD2-AS1 | lncRNA |
| AP001462.1 | lncRNA |
| AC009690.2 | lncRNA |
| MIR4435-2HG | lncRNA |
| METTL3 | m6A |
| WTAP | m6A |
| YTHDC2 | m6A |
| IGFBP1 | m6A |
| ZC3H13 | m6A |
| HNRNPA2B1 | m6A |
| YTHDF2 | m6A |
| IGFBP2 | m6A |
| RBMX | m6A |
| YTHDC1 | m6A |
| YTHDF1 | m6A |
| HNRNPC | m6A |
| METTL16 | m6A |
| FMR1 | m6A |
| METTL14 | m6A |
| IGFBP3 | m6A |
| RBM15 | m6A |
| YTHDF3 | m6A |
| RBM15B | m6A |

m^6^A, *N^6^*-methyladenosine; lncRNA, long non-coding RNA.
