# Supplementary material for: N 6 -Methyladenosine-Related Long Non-Coding RNAs Are Identified as a Potential Prognostic Biomarker for Lung Squamous Cell Carcinoma and Validated by Real-Time PCR
Source: Front Genet. 2022 Jun 3;13:839957. doi: 10.3389/fgene.2022.839957 (PMC9204524; doi:10.3389/fgene.2022.839957)
Supplement: Supplementary file 3 [file Table5.DOCX]

**Table S5** Multivariate Cox analysis of clinicopathological features and risk scores in the entire TCGA dataset

| id | HR | HR.95L | HR.95H | pvalue |
| --- | --- | --- | --- | --- |
| Age | 1.021902177 | 1.004921615 | 1.039169666 | 0.011269479 |
| Gender | 1.26588685 | 0.916629092 | 1.748220224 | 0.152302898 |
| Stage | 1.273898114 | 1.079149338 | 1.503792243 | 0.004238595 |
| riskScore | 1.740188758 | 1.217248222 | 2.487789147 | 0.002381014 |

HR, hazard ratio; TCGA, the cancer genome atlas.
