# Supplementary material for: N 6 -Methyladenosine-Related Long Non-Coding RNAs Are Identified as a Potential Prognostic Biomarker for Lung Squamous Cell Carcinoma and Validated by Real-Time PCR
Source: Front Genet. 2022 Jun 3;13:839957. doi: 10.3389/fgene.2022.839957 (PMC9204524; doi:10.3389/fgene.2022.839957)
Supplement: Supplementary file 4 [file Table3.DOCX]

**Table S3** Univariate Cox regression analysis to select 6 m^6^A-related lncRNAs with prognostic value

| gene | HR | HR.95L | HR.95H | pvalue |
| --- | --- | --- | --- | --- |
| AC138035.1 | 0.417055096 | 0.181730826 | 0.957102088 | 0.039073314 |
| HORMAD2-AS1 | 1.629705953 | 1.05686716 | 2.513032474 | 0.027086667 |
| AP001469.3 | 0.785106229 | 0.622424208 | 0.990308191 | 0.041134568 |
| AC243919.2 | 0.580425058 | 0.340787664 | 0.988572309 | 0.045255938 |
| PRC1-AS1 | 0.264488134 | 0.073742382 | 0.94862644 | 0.041261088 |
| AL122125.1 | 0.761490246 | 0.605167387 | 0.958193398 | 0.020112402 |

HR, hazard ratio
