# Supplementary material for: N 6 -Methyladenosine-Related Long Non-Coding RNAs Are Identified as a Potential Prognostic Biomarker for Lung Squamous Cell Carcinoma and Validated by Real-Time PCR
Source: Front Genet. 2022 Jun 3;13:839957. doi: 10.3389/fgene.2022.839957 (PMC9204524; doi:10.3389/fgene.2022.839957)
Supplement: Supplementary file 5 [file Table4.DOCX]

**Table S4** Univariate Cox analysis of clinicopathological features and risk scores in the entire TCGA dataset

| id | HR | HR.95L | HR.95H | pvalue |
| --- | --- | --- | --- | --- |
| Age | 1.017270257 | 1.000423492 | 1.034400716 | 0.04446745 |
| Gender | 1.196321694 | 0.867544266 | 1.649697487 | 0.274254892 |
| Stage | 1.255789216 | 1.06417234 | 1.481908988 | 0.007012503 |
| riskScore | 1.689811126 | 1.167725044 | 2.445320203 | 0.005397283 |

HR, hazard ratio
